# Persistence and Microevolution of *Pseudomonas aeruginosa* in the Cystic Fibrosis Lung: A Single-Patient Longitudinal Genomic Study

**DOI:** 10.3389/fmicb.2018.03242

**Published:** 2019-01-11

**Authors:** Irene Bianconi, Silvia D’Arcangelo, Alfonso Esposito, Mattia Benedet, Elena Piffer, Grazia Dinnella, Paola Gualdi, Michele Schinella, Ermanno Baldo, Claudio Donati, Olivier Jousson

**Affiliations:** ^1^Centre for Integrative Biology, University of Trento, Trento, Italy; ^2^Trentino Cystic Fibrosis Support Centre, Rovereto Hospital, Rovereto, Italy; ^3^Operative Unit of Clinical Pathology, Rovereto Hospital, Rovereto, Italy; ^4^Centro Ricerca e Innovazione, Fondazione Edmund Mach, San Michele all’Adige, Italy

**Keywords:** *Pseudomonas aeruginosa*, persistence, cystic fibrosis, genome evolution, antibiotic resistance

## Abstract

**Background:** During its persistence in cystic fibrosis (CF) airways, *P. aeruginosa* develops a series of phenotypic changes by the accumulation of pathoadaptive mutations. A better understanding of the role of these mutations in the adaptive process, with particular reference to the development of multidrug resistance (MDR), is essential for future development of novel therapeutic approaches, including the identification of new drug targets and the implementation of more efficient antibiotic therapy. Although several whole-genome sequencing studies on *P. aeruginosa* CF lineages have been published, the evolutionary trajectories in relation to the development of antimicrobial resistance remain mostly unexplored to date. In this study, we monitored the adaptive changes of *P. aeruginosa* during its microevolution in the CF airways to provide an innovative, genome-wide picture of mutations and persistent phenotypes and to point out potential novel mechanisms allowing survival in CF patients under antibiotic therapy.

**Results:** We obtained whole genome sequences of 40 *P. aeruginosa* clinical CF strains isolated at Trentino Regional Support CF Centre (Rovereto, Italy) from a single CF patient over an 8-year period (2007–2014). Genotypic analysis of the *P. aeruginosa* isolates revealed a clonal population dominated by the Sequence Type 390 and three closely related variants, indicating that all members of the population likely belong to the same clonal lineage and evolved from a common ancestor. While the majority of early isolates were susceptible to most antibiotics tested, over time resistant phenotypes increased in the persistent population. Genomic analyses of the population indicated a correlation between the evolution of antibiotic resistance profiles and phylogenetic relationships, and a number of putative pathoadaptive variations were identified.

**Conclusion:** This study provides valuable insights into the within-host adaptation and microevolution of *P. aeruginosa* in the CF lung and revealed the emergence of an MDR phenotype over time, which could not be comprehensively explained by the variations found in known resistance genes. Further investigations on uncharacterized variations disclosed in this study should help to increase our understanding of the development of MDR phenotype and the poor outcome of antibiotic therapies in many CF patients.

## Introduction

*Pseudomonas aeruginosa* is the most prevalent of all potential opportunistic nosocomial pathogens causing pulmonary and bloodstream infection with high mortality rates of up to 50% (Osmon et al., 2004). Cystic fibrosis (CF) patients infected with *P. aeruginosa* are exposed to increased morbidity and mortality (Lyczak et al., 2000; Chmiel and Davis, 2003). Progressive airway infection in CF subjects typically begins with recurrent *P. aeruginosa* infections that are often cleared by antibiotics and subsequently replaced by other strains that can establish permanent chronic infections in the respiratory tract (Folkesson et al., 2012). Long-term colonization of the CF airway is sustained by *P. aeruginosa* lineages which are clonal to the initially acquired strain (Bragonzi et al., 2009) and can persist in the airway of a patient for years and even decades (Jelsbak et al., 2007; Cramer et al., 2010).

Convergence of phenotypic changes among clinical isolates with a high level of initial phenotypic diversity within a single patient and between multiple strains from the same sample has been previously reported (Workentine et al., 2013; Jeukens et al., 2014; Caballero et al., 2015; Darch et al., 2015; Williams et al., 2015). Most common phenotypic changes during persistent infections include the conversion to a mucoid phenotype, loss of pigmentation, motility, secreted and cell-associated virulence factors, emergence of small colony variants (SCVs), use of alternative metabolic pathways, increased mutation rate, and the acquisition of multidrug resistance (Hogardt and Heesemann, 2010; Folkesson et al., 2012; Sousa and Pereira, 2014; Sommer et al., 2016).

A set of 24 genes in three different *P. aeruginosa* lineages have been reported to have undergone similar changes in their respective gene expression profiles (Huse et al., 2010), confirming the occurrence of convergent evolution in CF, in which different lineages tend to develop the same adaptive traits independently, due to the stressful CF lung environment. In another study (Marvig et al., 2015), the genomes of 474 longitudinal *P. aeruginosa* isolates collected from 34 CF patients for an average 5-year period were sequenced; they found 52 common genes mutated in all different lineages confirming that *P. aeruginosa* strains of various genetic backgrounds adapt to the CF airway following a convergent evolutive process. On the other hand, a high level of phenotypic diversity within a single patient and also between multiple isolates of the same sputum sample has also been reported (Huse et al., 2010; Folkesson et al., 2012). Although most *P. aeruginosa* infections have a clonal origin, persistent infections are characterized by an adaptive radiation process diversifying the initial clone in various morphotypes (Hogardt and Heesemann, 2010), and leading to the occurrence of subpopulations within the same host (Cullen and McClean, 2015).

As mentioned above, during chronic infection, the bacterium may develop a multidrug resistance (MDR) phenotype by the accumulation of pathoadaptive mutations or the acquisition of mobile elements by horizontal gene transfer (Breidenstein et al., 2011). Estimates indicate that 25–45% of adult CF patients are chronically infected with MDR *P. aeruginosa* within their airway (Lechtzin et al., 2006). Whole-genome sequencing (WGS) can help to point out potential molecular mechanisms of antibiotic resistance and has already proved to be able to successfully predict antimicrobial susceptibility in several pathogens (Stoesser et al., 2013; Zankari et al., 2013). Nevertheless, despite the availability of >2,000 *P. aeruginosa* genomes, knowledge on the evolution of genomic features associated with persistence and antimicrobial resistance is still incomplete.

In the present work, we sequenced the whole genome of 40 *P. aeruginosa* isolates from a single CF patient spanning an 8-year period. We analyzed the population in terms of clonality of the isolates, phylogenetic relationships, polymorphisms in the core genome and variations in the accessory genome. We also characterized the antibiotic resistance profile of the isolates and checked for mutations and variations in antibiotic resistance genes among the population. A better understanding of the evolutionary dynamics occurring during chronic airway infections in CF patients and of the genetic adaptation of strains to the CF lung environment should provide clues for preventive measures or novel therapies to control CF infections in the future.

## Materials and Methods

### Bacterial Strains

*Pseudomonas aeruginosa* strains were isolated from the sputum of a single adult male CF patient, aged 24 at the beginning of sampling. The patient carries the heterozygous CFTR mutations ΔF508 and G542X, and he was treated at the Trentino Regional Support CF Centre and the Operative Unit of Clinical Pathology (Hospital of Rovereto, Italy) (Supplementary Figure S1 and Supplementary Table S1).

A total of 40 *P. aeruginosa* strains were isolated over an 8-year period (2007–2014). To evaluate both the suitability of the material from the lower respiratory tract of the patient (sputum) and the putative pathogens present, a microscopic examination was carried out using Bartlett’s criteria. Sputum samples were fluidized with the addition of dithiothreitol and plated on blood agar and MacConkey agar plates. *Pseudomonas aeruginosa* was identified using the API ID32GN kit (bioMérieux, Bagno a Ripoli, Italy). Strains from the same sputum samples showing a different colony morphology were collected and characterized separately (Bianconi et al., 2016; D’Arcangelo, 2017).

### Antimicrobial Susceptibility Testing

MIC values for all 40 strains were determined by reference broth microdilution using TREK Sensititre custom panel ITGNEGF (Thermo-Fisher, Waltham, MA, United States). The following 12 antibiotics belonging to nine different classes were tested: amikacin, gentamicin, imipenem, meropenem, doripenem, ceftazidime, cefepime, ciprofloxacin, levofloxacin, fosfomycin, colistin, and a combination of piperacillin/tazobactam.

### Mucoid Phenotype

Isolates were inoculated on Pseudomonas Isolation Agar (BD, Franklin Lakes, NJ, United States) for 48–72 h at 37°C. Strains producing alginate were considered positive (Bragonzi et al., 2006).

### Biofilm Formation Assay

Biofilm formation was assessed by the crystal violet staining assay with minor modifications (O’Toole, 2011). Briefly, pellets of overnight cultures were collected by centrifugation and re-suspended in M63 liquid medium (Amresco, Solon, OH, United States) to a final OD_600_ of 0.005 and aliquoted in a 96-well U-bottom microtiter plate (Costar, Washington, DC, United States). After 24 h of static incubation at 37°C, supernatants were removed, and OD_600_ was measured. Cells attached to the microtiter wells were washed and stained with an aqueous solution of 0.1% crystal violet. Cells were washed and resuspended in 30% acetic acid. OD_550_ of resuspended biofilm was determined and normalized to the OD_600_ of the corresponding planktonic cells. The assay was repeated at least three times per isolate.

### DNA Extraction, Genome Sequencing, and Assembly

The genome sequences of the isolates were previously published under accession numbers MAUO00000000 – MBMR00000000 (Supplementary Table S2) (Bianconi et al., 2016).

Genomic DNA was extracted from overnight cultures in LB Broth (Oxoid, Sigma-Aldrich, Darmstadt, Germany) at 37°C with continuous shaking, using the DNeasy Blood and Tissue Kit (Qiagen, Hilden, Germany) following the manufacturer’s instructions for Gram-negative bacteria. Sequencing libraries were prepared using the Nextera XT DNA Library Preparation Kit (Illumina, San Diego, CA, United States) with default settings and sequenced on the Illumina MiSeq platform. *De novo* assembly of draft genomes was carried out using SPAdes version 3.1.0 (Bankevich et al., 2012), with the following options: –careful on and k-mer sizes set as 21,33,55,77,99,127. To further improve the quality of the assemblies, raw reads were mapped on the contigs using Bowtie2 (Langmead and Salzberg, 2012), and contigs with less than three reads mapping and/or with coverage below one were removed. Draft genomes were reordered using Mauve (Darling et al., 2010) with *P. aeruginosa* DK2 strain sequence used as a reference.

### Genotyping, Genome Annotation, Pangenome, and SNPs Analysis

MLST 1.8 (Larsen et al., 2012) was used for genotyping of the population from *de novo* assembled genomes. An eBURST analysis (eBURST V3, Feil et al., 2004) was carried out to estimate the relatedness of Sequence Types (STs). *In silico* determination of the *O*-antigen was performed using the *Pseudomonas aeruginosa* serotypes (PAst) script^1^. Genome annotation was performed with Prokka automatic annotation tool v1.11 (Seemann, 2014), and analysis of the core and accessory genomes was carried out using Roary with default settings (Page et al., 2015). An accessory genome presence matrix was created from the standard Roary output file and isolates’ values were plotted ordered by isolation date with the ggpubr package inside R/Bioconductor environment^2^. To assess the number of gene acquisition/loss events, all acquired genes (*n* = 542) were pooled to a gene dataset composed by the accessory genes of the first isolated strains TNCF_3 and TNCF_4M (*n* = 624); each isolate was compared with these two datasets. The barplot was generated using the ggpubr package as above.

SNPs were identified using Snippy^3^ (Seemann, 2015). This software infers polymorphisms at the nucleotide level by aligning the unassembled reads to a reference genome (in this study we used *P. aeruginosa* DK2) and it calls the software SnpEff to annotate and predict the effect of each mutation (Cingolani et al., 2012), dividing them into low (e.g., synonym mutations), moderate (e.g., inframe deletion/insertion) and high (e.g., stop gained).

### Plasmids and Genomic Islands Prediction

To detect the presence of plasmids from NGS data, the software PlasmidSeeker was used (Roosaare et al., 2018). The algorithm allows the identification of plasmids using the information of plasmid k-mer coverage, which is expected to be higher than that of the genome. For each set of reads we used the respective assembled genome. In addition, we performed two separate analyses using the reference genomes of PAO1 and PA14. The plasmids k-mer database consists of >8,500 known plasmids from several species, 14 of which are specific for *P. aeruginosa*.

The occurrence and distribution of genomic islands (GIs) were inferred using IslandViewer4 (Bertelli et al., 2017). Prokka output files in GenBank format were uploaded to the IslandViewer web server. To obtain a set of homologous protein clusters (PCs), the proteins found by IslandViewer were clustered using CD-HIT (Fu et al., 2012), with both coverage and identity percentage set at 95%. A genome map of the distribution of the islands in each isolate was drawn using the package genoplotR. Also, PCs were mapped against a custom dataset of 23 previously characterized GIs using tblastn. Ubiquitous PCs that could not be assigned to any of the known GIs were searched on the whole non-redundant NCBI protein database using blastp, restricting the analysis to *P. aeruginosa* sequences. To determine the occurrence of a phylogenetic signal in the distribution of non-ubiquitous PCs, a phylo.d analysis (R-package caper) (Fritz and Purvis, 2010) was performed.

### Type II Toxin–Antitoxin (TA) Systems Detection

Putative type II toxin–antitoxin (TA) loci were identified by using the online server TAfinder^4^ (Shao et al., 2011; Xie et al., 2018). Newly assembled genomes were previously annotated with the online tool CDSeasy^5^. Output GenBank files were uploaded in TAfinder and analyzed using default parameters.

### Phylogenetic Tree Construction

A rooted phylogenetic tree, based on the distribution of SNPs in the core genome, was then obtained using BEAST V1.8.3 (Drummond et al., 2012). A Markov Chain Monte Carlo chain was run for 9 × 10^8^ steps using the uncorrelated relaxed clock model (Drummond et al., 2006) with lognormal distribution and gtr substitution model with gamma-distributed rates, four categories, shape parameter α. After a burn-in of 4 × 10^8^ steps, trees were recorded every 10.000 steps, and the consensus tree was determined using TreeAnnotator. To evaluate the amount of recombining sites, the phylogenetic tree, together with the alignment of core genes obtained by Roary, were analyzed using clonalframeML (Didelot and Wilson, 2015).

### Detection of Phylogenetic Signal and Molecular Evolution Analysis

The phylo.d function within the R package was used to measure the strength of phylogenetic signal in binary traits^6^. Two simulated null models were used to standardize the phylogenetic signal: phylogenetic randomness and Brownian threshold.

Genes with multiple SNPs were initially considered to be under non-neutral selection. This statement was further verified with the following procedure: (i) the alignment of each homologous gene with reference sequence was exported in FASTA format; (ii) the dN/dS ratio was determined using codeml as implemented in PAML package (Yang, 2007) and the likelihood of four different models (two neutral or nearly neutral, M1a and M7, respectively, and the two corresponding positive selection models M2a and M8) was determined; (iii) the likelihood ratio test, using as null model the neutral one, was performed to determine which model better fitted the observed data (Anisimova and Gascuel, 2006; Yang, 2006). The procedure was implemented in a conservative way to detect non-neutral selection in highly similar sequences, using as null model the neutral one.

## Results

### Whole-Genome Sequencing and *in silico* MLST Genotyping

In this study, we sequenced 40 *P. aeruginosa* isolates from a CF patient over an 8-year period. The average number of contigs per genome was 101 (range 53–356). Mean size of the draft genomes was 6.6 Mb (range 6.545–6.653). Mean G+C content was 66,28% and the genomes encoded for an average of 6,151 putative ORFs. Details of the genomes obtained are provided in Table 1 and preliminarily described in Bianconi et al. (2016).

**Table 1:** Genomic features and statistics of the 40 *P. aeruginosa* CF isolates.

Values	Assembly statistics						Annotation statistics	
	# Read pairs	Avg.cov	# Contigs	N50	GC%	TOT length	CDS	tRNA
Minimum	156677	4	53	31	66.27	6545	6052	53
Average	581968	22	101	180	66.28	6633	6151.4	59
Median	517366	21	86	177	66.28	6635	6151.5	60
Maximum	1226553	42	356	378	66.36	6653	6219	66

**FIGURE 1:**
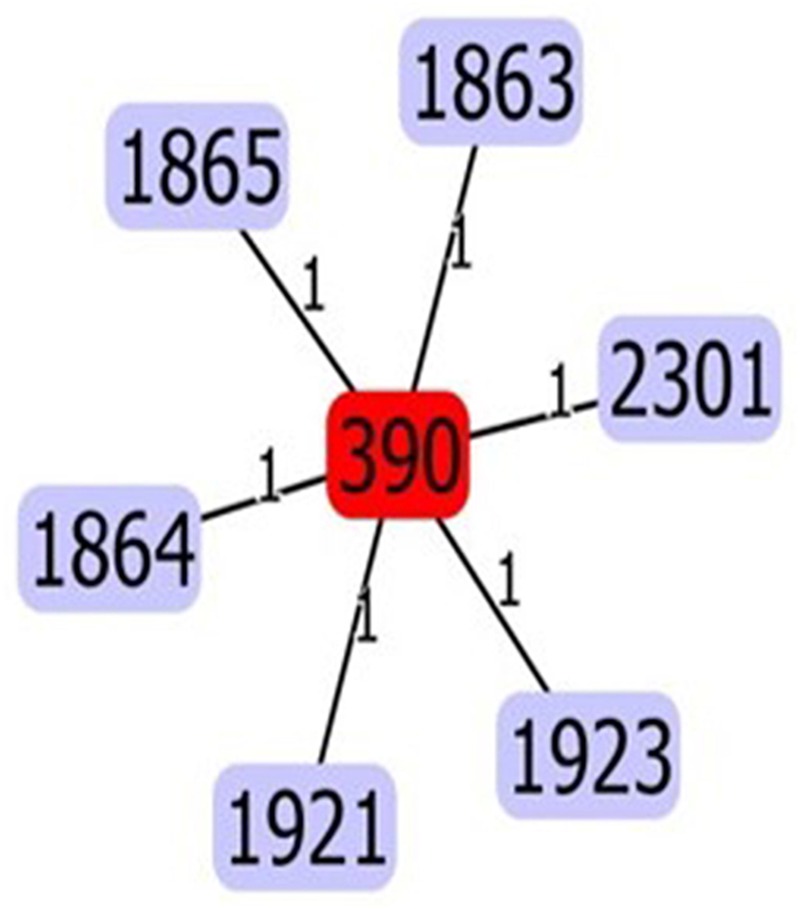
eBURST analysis of the sequence types found in the *P. aeruginosa* population. The five variants (ST1863, ST1864, ST1865, ST1921, ST2301, and ST1923) differed from ST390 at a single locus. Variants ST1863, ST1864, ST1923 were discovered in the present study.

*In silico* MLST analysis revealed a population dominated by a previously characterized Sequence Type (ST390, 30 isolates), and three new ST variants (Figure 1): ST1864 (six isolates: TNCF_16, TNCF_85, TNCF_88M, TNCF_101, TNCF_133_1, TNCF_151M), ST1923 (three isolates: TNCF_155_1, TNCF_165, TNCF_176) and ST1863 (one isolate: TNCF_69). ST390 (allelic profile: acs39-aro5-gua1-mut3-nuo4-pps46-trp56) was previously detected in six isolates: in a sputum sample from a CF patient (Canada, in 2004), in an environmental sample (France, in 2009), and in four unspecified isolates^7^. The novel variants were deposited in the PubMLST database^8^.

### Phylogenetic Analyses

**FIGURE 2:**
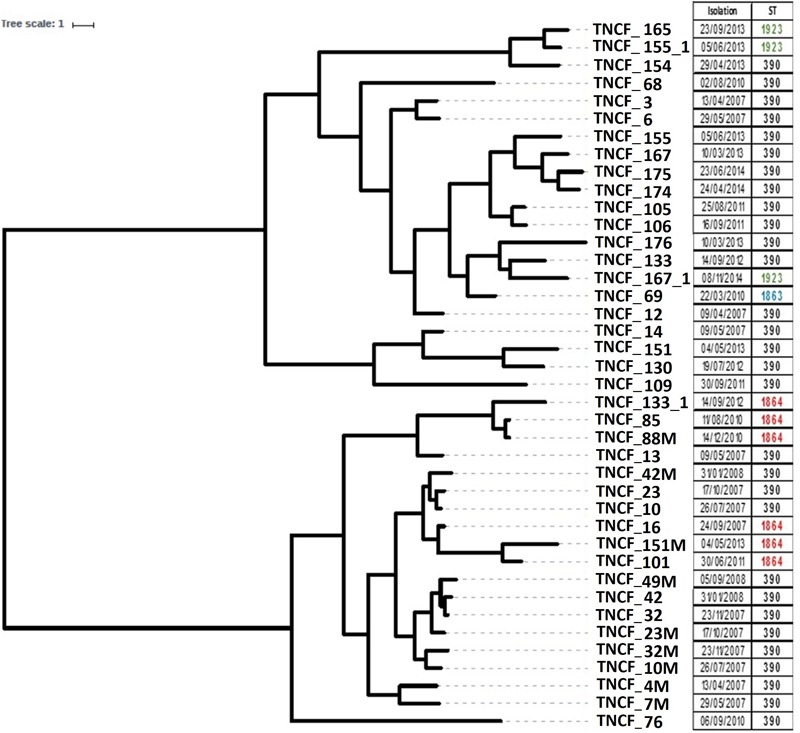
Rooted phylogeny of the *P. aeruginosa* population. The tree was built on core genome SNPs obtained using BEAST V1.8 with 10 independent runs of 10^9^ steps using the relaxed clock model. Different strains isolated from the same sputum sample share the same isolation date. The right panel reports the date of sampling and the Sequence Type of each isolate.

To reconstruct the evolutionary history of the lineage and to examine the relationships between the isolates we analyzed the distribution of SNPs in the core genome using BEAST. The tree topology (Figure 2) indicates that the population is composed of two main clusters separated by a sizeable basal split: the first one comprised nearly all the early isolates (13 out of a total of 17 isolates sampled between 2007 and 2008), whereas the majority of the late isolates branched within the second cluster (17 out of a total of 23 isolates sampled between 2010 and 2014). The most recent common ancestor of the former clade is dated by BEAST in 1988 ± 19.9 years, and in 1992 ± 14.8 years for the latter, while the date of the divergence of the two clades was estimated to be 1979 ± 21 years. Such high standard deviations thus do not allow to infer divergence times in the population. Regardless of their placement in the phylogenetic tree, all late isolates had longer branches compared to early ones, indicating a more substantial genomic diversity of the formers and/or a different evolutionary rate between the two groups of isolates. The six ST1864 isolates appear to be polyphyletic and fall in the cluster with ST390 early isolates, while the three ST1923 isolates and the ST1863 one group in the cluster with the late ST390 isolates sampled between 2010 and 2014.

Excluding homoplasic sites with ClonalFrameML (Supplementary Figure S2), a number of branches collapsed, suggesting that most of the variability within the population is due to horizontal transfer and/or convergent evolution.

### Core and Accessory Genome Analyses

**FIGURE 3:**
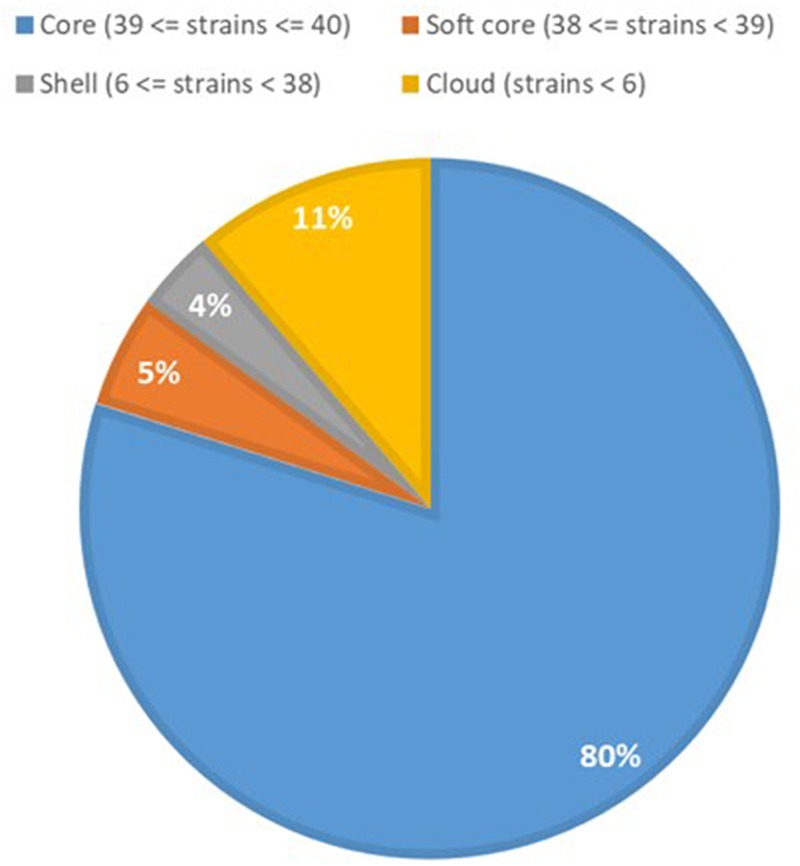
Core and accessory genome proportions in the *P. aeruginosa* population. Genomic portions present in 38 or 39 isolates out of 40 (softcore) were considered as core genes in the text due to the draft assembly of the genomes.

**FIGURE 4:**
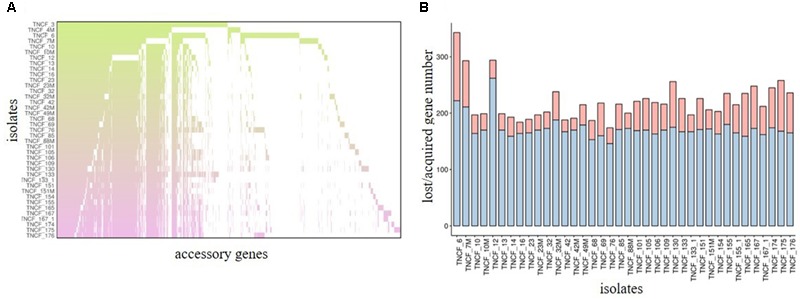
Analyses of the accessory genome. **(A)** Distribution of accessory genes in the population. Each row represents an isolate and each column represents a gene (gene labels not shown). The gradient represents the time (in days) starting from the isolation date of the first two strains (TNCF_3 and TNCF_4M). **(B)** Loss or acquisition of accessory genes in the population. Barplots represent the acquired/lost genes (in red and blue, respectively) for each isolate in comparison with the two first isolates TNCF_3 and TNCF_4M.

Previous studies have shown that the genomes of *P. aeruginosa* isolates are usually highly conserved (Klockgether et al., 2011, 2013). Analyses of the core and accessory genome of the population confirmed the high homogeneity of these strains revealed by *in silico* MLST. The pan-genome of the population comprised 7,035 genes, with nearly 85% of the genes shared between all the isolates (5,972 genes). Among these, 5608 were core genes present in 39–40 isolates, and 364 were soft core genes found in at least 38 isolates (Figure 3). A total of 1,166 genes (16%) belonged to the accessory genome (Figure 4A). Among these, 624 were shell genes present in the initial isolates (TNCF_3 or TNCF_4M), while 542 were acquired in the course of time (2,766 days). Among the latter genes, 320 were found to be present only in one isolate (TNCF_6). The average number of accessory genes per genome was 500, with TNCF_12 being the isolate with the smallest accessory genome (394 genes) and TNCF_6 the one with the largest one (523 genes), consistent with the greater length of its genome. In order to get insight into the dynamics of acquisition/loss of accessory genes, we assessed for each isolate the number of genes belonging to each class. As shown in Figure 4B, the general trend in the microevolution of this population leans toward a higher number of gene loss events compared to gene acquisition. The accessory genomes mapped on the phylogenetic tree (Supplementary Figure S3) showed an extensive collapse at the root, and the presence of three major lineages: one includes 31 out of 40 isolates (the bulk of the population); a second lineage comprises isolates TNCF_23, TNCF_10 and TNCF_42M, which formed a monophyletic group also in the SNP-based tree; the third lineage includes TNCF_16, TNCF_7M, TNCF_4M, TNCF_10M, TNCF_32M, TNCF_151M. TNCF_6 also resulted in being the strain with the most diverse accessory genome, in agreement with its high number of unique regions.

Clustering of isolates based on the composition of the accessory genome (Supplementary Figure S4) revealed a group of 11 highly similar genomes comprising both early and late isolates, eight belonging to the dominant ST390 type (TNCF_10, TNCF_13, TNCF_23, TNCF_23M, TNCF_32, TNCF_42, TNCF_42M, TNCF_49M) and three belonging to the derivate ST1864 (TNCF_16, TNCF_88M and TNCF_133_1). Accordingly, the number of accessory genes in each genome of this cluster was remarkably homogeneous, ranging from 488 to 511 genes in TNCF_16 and TNCF_10, respectively.

### Identification of Plasmids and Genomic Islands

The analyses performed using PAO1, PA14 and each assembled genome as reference detected only one plasmid, belonging to the pKLC102 family, which was ubiquitous in the population with the exception of isolate TNCF_12.

**FIGURE 5:**
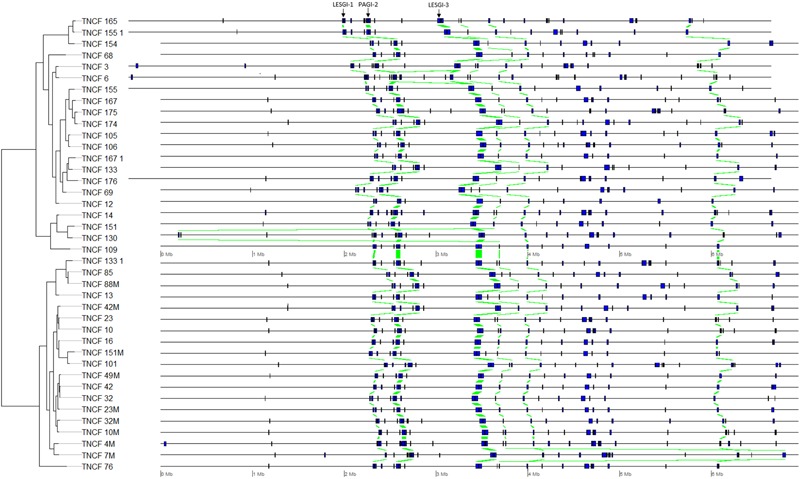
Genomic map showing the position of each genomic island on each genome. Homologous regions are highlighted in green.

The distribution of PCs was strongly skewed toward the two extremes, i.e., most of the PCs were either ubiquitous or isolate-specific; 87 PCs, belonging to six GIs, were ubiquitous in the population (Figure 5). The tblastn analysis performed on our custom dataset of known GIs detected four PCs (with identity > 80% and e-value < 4.7 e^80^) putatively belonging to the island LESGI-1 (Winstanley et al., 2009), two PCs (id > 85%, e-value < 1.7 e^46^) were assigned to LESGI-3 (Winstanley et al., 2009), and three PCs (id > 90%, e-value < 5.3 e^80^) were assigned to the island PAGI-2 (Klockgether et al., 2007).

Twenty-one ubiquitous PCs, belonging to the three yet unidentified GIs found in the collection, were mapped against the non-redundant NCBI database restricting the search to *P. aeruginosa*. Most PCs matched with hypothetical proteins, and only six PCs matched with the following annotated proteins: YqaJ-like viral recombinase domain cl09232 (WP_031641785), recombination protein RecT (WP_019682741), resolvase (EJY58266), serine recombinase (WP_070330427), DNA ligase [NAD(+)] LigA (WP_003114675), *N*-acetyltransferase (WP_003096122).

### Type II Toxin–Antitoxin Systems

Most genes belonging to the TA systems were found to be ubiquitous in the population. Non-ubiquitous TA genes belonged to domains COG1733 (found in 37 isolates), pfam13420 (38 isolates), pfam00392 (39) and PRK10151 (which was found only in one isolate, TNCF_7M). TA families Abr-like (COG4456 domain), vapC (cd009981 domain) and RHH-like were present in nearly all isolates (39). Genes belonging to the Xre-like family were the most abundant with at least four genes in each genome (Supplementary Figure S5).

The total number of TA genes found in the isolates ranged from 30 (in TNCF_12) to 36 (in TNCF_101), and the average number of genes was 34. There was no difference among early and late isolates in terms of presence or abundance of TA families or domains, (Supplementary Figure S6).

### Variants and SNPs Analysis

We looked at the distribution of variants in the core genome of the population, using DK2 as a reference, as this strain resulted in being the closest relative to this population (data not shown). Data corresponding to insertions/deletions was not considered as the draft nature of the genomes could bias the output of the program. Each isolate contained a total of 27,249 ± 3,300 single nucleotide variations. In particular, variants per genome ranged from 12,983 to 29,744 in the isolates TNCF_7M and TNCF_165, respectively, with a median number of 28,668 nucleotide variations per genome (as in TNCF_23M and TNCF_32) (Supplementary Table S2).

To investigate polymorphisms with a presumable impact on the adaptive process of the isolates, we extracted non-synonymous polymorphisms classified as high impact variants (i.e., variants resulting in protein truncation, loss of function or triggering non-sense-mediated decay). Considering the complete genome sequence of the isolates (i.e., both core and accessory genomes), the number of high impact variations considerably varied in the population, ranging from six in the early isolate TNCF_7M to 167 in the late isolate TNCF_175.

To determine whether the majority of variants fall into few functional categories or are randomly distributed among all of them, we performed a Cluster of Orthologous Groups (COG) analysis, wherein we assigned the genes in which high-impact variants were found to their respective COG categories. The percentage of genes assigned to each COG is shown in Supplementary Figure S7. Mutations occurred primarily in genes involved in transcription (8%), followed by amino acid transport and metabolism, inorganic ion transport and metabolism, signal transduction mechanisms and general functions (7% each). A large proportion of genes (28%) were not assigned to any COG category but were classified as genes with unknown function. The remaining mutated genes were distributed into all functional groups, with proportions reflecting those found in the analysis of whole genomes.

### Polymorphisms in the Core Genome of the Population

We then extended the analysis to variants in the core genome with high and moderate impact (non-disruptive variants that might change protein effectiveness). These ranged from 132 (TNCF_7M) to 394 (TNCF_167, TNCF_175) per genome, with a mean of 270 (Supplementary Table S2). Moreover, an analysis of the distribution of variants in the population (Supplementary Figure S8) revealed a cluster of 13 isolates (TNCF_10, TNCF_13, TNCF_16, TNCF_23, TNCF_23M, TNCF_32, TNCF_42, TNCF_42M, TNCF_49M, TNCF_85, TNCF_88M, TNCF_101, TNCF_133_1) with a high similarity in their variants pattern. One isolate, TNCF_7M, stands out in the distance matrix, probably due to the lowest number of SNPs present in the genome.

The analysis also highlighted the diverse subpopulations putatively derived from the differentiation of a first infecting strain, e.g., the isolates belonging to the ST1864 cluster, which showed a marked similarity, with the only exception of the isolated TNCF_151M, which was quite divergent compared to the other strains.

Eight genes belonging to the core genome carried high-impact variants. Interestingly, four of them have unknown function. The gene encoding respiratory nitrate reductase subunit gamma *nail* carried high impact variants in three closely related isolates, one early, TNCF_14, and two lates, TNCF_130 and TNCF_151. Isolates TNCF_133 and TNCF_167_1 had high impact variants of two genes encoding a polyprotein signal peptidase *lspA* and a hypothetical protein, and two hypothetical proteins, respectively. Other genes with high-impact variants were those encoding the ABC transporter-binding protein *aaltP* in TNCF_3, the copper resistance protein A precursor *pcoA* in TNCF_175 and a gene with unknown function in TNCF_174.

Within the whole population, a total of 675 different genes carried mutations with moderate impact within the core genome and the distribution of these variants was consistent with population structure (Supplementary Figure S8). Focusing on the moderate impact polymorphisms in the core genome, we found 20 genes with three or more of such variations in the population (Supplementary Table S3), which might be indicative of loci under selective pressure (Table 2). Remarkably, 8 of them were genes with a putative or unknown function. The pyoverdine biosynthesis gene *pvdL* carried a total of five moderate-impact SNPs in all 40 genomes analyzed, as well as the DNA gyrase subunit B *gyrB* with five variants in 36 genomes. Remarkably two amino acid substitutions, E_468_-D and S_749_-P, were found in isolates resistant to fluoroquinolones. The topoisomerase IV subunit B *parE* gene showed three different variations within the population, including R_30_-C and H_59_-R in the late MDR isolates. The two-component regulator system signal sensor kinase *pmrB* gene showed three different variations among the population; *glnE* and *rsme (yggJ)* carried three different variants, while *cbrA* and *gltB, nagE, aruF* and *pilL* had three moderate variants in five, four, and three different genomes, respectively.

**Table 2:** Core genes with the highest number of SNPs in the population.

Gene name	Product name	Variations (n.)
***gyrB***	**DNA gyrase subunit B**	**5**
*pvdL*	Non-ribosomal peptide synthase, pyoverdine biosynthesis	5
15595766 ^∗^	Hypothetical protein	4
*aruF*	Subunit I of arginine N2-succinyltransferase = ornithine N2-succinyltransferase	3
*ftsJ*	Cell division protein FtsJ	3
***chpA***	**Component of chemotactic signal transduction system**	**3**
***gidA***	**Glucose-inhibited division protein A**	**3**
*glnE*	Glutamate-ammonia-ligase adenylyltransferase	3
***gltB***	**Glutamate synthase subunit alpha**	**3**
***nagE***	***N*-Acetyl-D-glucosamine phosphotransferase system transporter**	**3**
***parE***	**DNA topoisomerase IV subunit B**	**3**
*pmrB*	Two-component regulator system signal sensor kinase PmrB	3
*cbrA*	Two-component sensor CbrA	3
15595416 ^∗^	Probable aldehyde dehydrogenase	3
15595440 ^∗^	Probable transcriptional regulator	3
15596248 ^∗^	Probable transporter	3
**15596746 ^∗^**	**Probable cation-transporting P-type ATPase**	**3**
**15597654 ^∗^**	**Hypothetical protein**	**3**
**15599121 ^∗^**	**Probable major facilitator superfamily (MFS) transporter**	**3**
15597414 ^∗^	Hypothetical protein	3

*The 20 genes listed above belong to the core genome and have at least three different polymorphisms with a moderate impact on the encoded protein according to Snippy analysis (Seemann). Genes under non-neutral pressure are in bold. ^∗^ Corresponding gene accession number in the reference strain PAO1, when the gene name is not available.*

We found that at least three genes, *gyrB, parE*, and *pmrB*, involved in the resistance to different classes of antibiotics carried mutations potentially affecting the protein function (Supplementary Table S2).

Among the 20 most variable genes (with at least three variations within the population) selected for the analysis of adaptive evolution, nine resulted in being under non-neutral pressure (namely *gyrB* and *parE, pilL, nagE, gltB, gidA*, and three genes encoding hypothetical proteins) (Table 2). For 10 of them, the neutral (null) hypothesis could not be rejected. For one gene, *pvdL*, it was not possible to perform the test, because its sequence was incomplete, probably due to the draft nature of the genomes. All genes for which the likelihood ratio test rejected the null hypothesis had an omega (dN/dS) > 1, suggesting they are under positive selection.

### Phenotypic Analyses

The mucoid phenotype, a characteristic marker of adaptation to the CF lung, was present in 12 strains (30%), mainly early and intermediate isolates, while 17 isolates (42.5%) produced a detectable amount of biofilm (Supplementary Table S4).

For the mucoid phenotype, there was a significant inverse correlation (ρ = -0.407, *p* = 0.009) with the time of isolation, whereas Spearman’s coefficient indicated no significant correlation (ρ = 0.202, *p* = 0.211) between biofilm formation and time of sampling. A significant correlation between alginate production and biofilm formation was observed (Supplementary Table S5).

### Antibiotic Resistance Profiles

**FIGURE 6:**
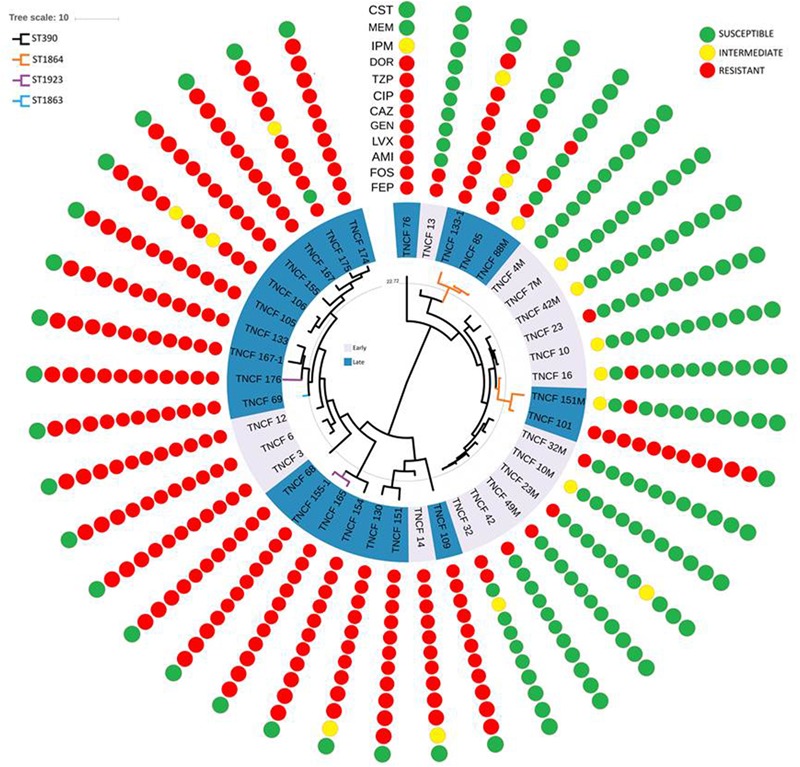
Rooted phylogeny of the *P. aeruginosa* population with antibiotic susceptibility profiles. Resistance and susceptibility were determined according to the EUCAST clinical breakpoint tables. Resistant, intermediate and susceptible isolates are highlighted in red, yellow, and green, respectively. AMI, amikacin; GEN, gentamicin; IPM, imipenem; MEM, meropenem; DOR, doripenem; CAZ, cefepime; FEP, ceftazidime; CIP, ciprofloxacin; LVX, levofloxacin; FOS, fosfomycin; CST, colistin; TZP, piperacillin/tazobactam.

Antibiotic susceptibility tests were performed for all isolates of the population (Figure 6). Twelve different antibiotics belonging to nine classes were tested (see the section “Materials and Methods”). All the isolates were susceptible to colistin, whereas nearly all the early isolates were susceptible to almost all the antibiotics tested. On the other hand, resistant and multidrug-resistant (MDR) phenotypes dramatically increased over time in the persistent population (Supplementary Figure S9).

The vast majority of the 17 strains isolated in 2007 and 2008 were susceptible to nearly all the antibiotics tested, in particular, TNCF_4M was found susceptible to all the antibiotics, while TNCF_7M, TNCF_10, TNCF_10M, and TNCF_42M had an intermediate resistance profile only to cefepime. Four strains were found to have an MDR phenotype: TNCF_3, TNCF_6, TNCF_12, and TNCF_14 were resistant to all antibiotics tested with the exception of colistin.

Among 23 strains isolated between 2010 and 2014, only two of them, TNCF_88M and TNCF_151M, both belonging to ST1864, did not show an MDR phenotype: TNCF_88M, isolated on December 2010, was susceptible to eight antibiotics, with an intermediate profile to cefepime and resistant to ciprofloxacin, levofloxacin, fosfomycin, and piperacillin/tazobactam; whereas TNCF_151M, isolated on May 2013, was found to be susceptible to 10 antibiotics tested, with an intermediate profile to Cefepime and resistant only to amikacin.

The exacerbation of patient’s lung conditions started approximately at the time of predominance of MDR, and biofilm-forming isolates (Supplementary Figures S1, S9; Supplementary Table S4). In the latest stages of infection, the selected antibiotic therapy consisted of a combination of glycopeptides, aminoglycosides, and carbapenems.

As a mucoid phenotype and biofilm formation are often associated with an increase of antibiotic resistance, Spearman’s correlation coefficient was applied to calculate the correlation between the three phenotypes (Supplementary Table S4). MDR was negatively significantly correlated with mucoidy and positively correlated with biofilm formation.

Genomic analyses of the population showed a correlation between the evolution of antibiotic resistance profiles, MLST sequence types, and phylogenetic relationships (Figure 6). We further investigated the MDR isolates in comparison to the susceptible ones, looking specifically at the coding sequences of *P. aeruginosa* genes involved in antibiotic resistance and susceptibility. As described above, non-synonymous mutations were found in *gyrB, parE* and *pmrB*; additional variants present in the MDR isolates compared to the susceptible ones were: ΔC_437_ in the outer membrane porin gene *oprD* leading to a frameshift mutation in nearly all MDR isolates, P_51_-S in MexG and N_170_-S in the topoisomerase IV subunit A ParC (Table 3 and Supplementary Table S6).

**Table 3:** Variations in genes involved in antibiotic resistance.

Gene	Nt mutation	Variation	Isolates
*gyrB*	G_1404_-T	E_468_-D	TNCF_3, TNCF_6, TNCF_12, TNCF_13, TNCF_14, TNCF_68, TNCF_69, TNCF_76, TNCF_85, TNCF_88M, TNCF_101, TNCF_105, TNCF_106, TNCF_109, TNCF_130, TNCF_133_1, TNCF_151, TNCF_155, TNCF_165, TNCF_167, TNCF_167_1, TNCF_174, TNCF_175, TNCF_176
	T_2245_-C	S_749_-P	TNCF_10, TNCF_10M, TNCF_23, TNCF_23M, TNCF_32, TNCF_32M, TNCF_42, TNCF_42M, TNCF_49M
*mexG*	C_151_-T	P_51_-S	TNCF_3, TNCF_12, TNCF_68, TNCF_76, TNCF_85, TNCF_88M, TNCF_105, TNCF_106, TNCF_109, TNCF_130, TNCF_133, TNCF_151, TNCF_155, TNCF_165, TNCF_167, TNCF_167_1, TNCF_174, TNCF_175, TNCF_176
*oprD*	ΔC_437_	frameshift	TNCF_3, TNCF_6, TNCF_12, TNCF_68, TNCF_69, TNCF_101, TNCF_105, TNCF_106, TNCF_133, TNCF_155, TNCF_165, TNCF_167, TNCF_167_1, TNCF_174, TNCF_175, TNCF_176
*parC*	A_509_-G	N_170_-S	TNCF_3, TNCF_12, TNCF_14, TNCF_68, TNCF_76, TNCF_101, TNCF_105, TNCF_106, TNCF_109, TNCF_130, TNCF_151, TNCF_155, TNCF_165, TNCF_167, TNCF_167_1, TNCF_174, TNCF_175, TNCF_176
*parE*	T_88_-C	R_30_-C	TNCF_69, TNCF_105, TNCF_106, TNCF_133, TNCF_155, TNCF_155_1, TNCF_165, TNCF_167, TNCF_167_1, TNCF_174, TNCF_175, TNCF_176
	G_176_-A	H_59_-R	TNCF_69, TNCF_105, TNCF_106, TNCF_133, TNCF_155, TNCF_155_1, TNCF_165, TNCF_167, TNCF_167_1, TNCF_174, TNCF_175, TNCF_176

*Nt, nucleotidic.*

## Discussion

*Pseudomonas aeruginosa* undergoes a characteristic evolutionary adaptation during chronic infection in the CF lung. Previous genomic studies have revealed extensive strain genetic variability within patients, and adaptation to the CF lung environment is recognized to play a crucial role in the diversification of *P. aeruginosa* (Winstanley et al., 2016).

The occurrence of a dominant Sequence Type in the population together with a small number of closely related genotypes indicates that all isolates likely belong to the same clonal lineage and possibly evolved from a single ancestral colonizing strain. This is in contrast with what has been previously reported in other studies, in which either different lineages coexist within CF patients (Feliziani et al., 2014; Caballero et al., 2015; Williams et al., 2015) or a first infecting lineage is replaced by a new one (Rau et al., 2010; Bianconi et al., 2015). In this patient, a single dominant clonal lineage apparently persisted in the airways for at least 8 years, allowing us to perform a detailed study of the microevolution and adaptive process of the bacterium in the course of chronic infection.

We report the presence of two main lineages in the population, with one of them being the most prevalent in the latest stage of infection (Figure 1). Remarkably, this cluster comprised most multi-drug resistant isolates. Although most of the *P. aeruginosa* infections have a clonal origin, persistent infections are characterized by an adaptive radiation process leading to a marked diversification of the bacterial clones (Cullen and McClean, 2015). The adaptive process most likely occurs independently in the different individuals of the bacterial population, resulting in the occurrence of clonal subpopulations within the same host (Breidenstein et al., 2011). We have previously characterized genotypically and phenotypically an extensive collection of *P. aeruginosa* clinical strains isolated from chronic and acute infections (Ballarini et al., 2012). One acute infection isolate (VrPa97), from a wound swab and isolated at the hospital of Verona, Italy, was found to belong to the same genotype (ST390) as that of the isolates analyzed in the present work, thus supporting the idea that very closely related strains can cause acute infections and evolve into highly adapted phenotypes after selection and persistence in the CF lung environment.

A number of comparative genomic studies of *P. aeruginosa* have shown that most inter-strain diversity is due to the presence of regions of genomic plasticity (RGPs) or GIs often acquired by horizontal gene transfer (Mathee et al., 2008; Kung et al., 2010; Pohl et al., 2014). Plasmid pKLC102 plus six GIs were detected in the population. pKLC102 was previously found in strains of very different origins; this highly motile integrative and conjugative element is almost ubiquitous in *P. aeruginosa* (Klockgether et al., 2007). It was found to coexist in both episomal and chromosome-integrated forms in *P. aeruginosa* clone C strains (Klockgether et al., 2004). Three GIs could not be assigned to any previously characterized island, whereas the other ones could be assigned to LESGI-1, PAGI-2, and LESGI-3. LESGI-1 and LESGI-3 were first identified and characterized in the epidemic *P. aeruginosa* strain LESB58 (Winstanley et al., 2009). LESGI-1 contains phage- and transposon-related genes, in addition to coding sequences showing similarity with proteins of non-pseudomonad species (Winstanley et al., 2009). LESGI-3 island has a mosaic structure and presents a significant homology to PAGI-2, PAGI-3, PAGI-5, and PAPI-1 (Winstanley et al., 2009; Silby et al., 2011). To date, it was described as a unique island of the LESB58 strain, since its particular structure was not found in other strains (Fothergill et al., 2010; Jani et al., 2016). However, even if three PCs showed high identity with both LESGI-3 and PAGI-2, two additional ones with an exclusive assignment to LESGI-3 were found in a different genomic region. It is, therefore, more straightforward to assign the first genomic region to PAGI-2 and the second genomic region, located about 1 Mb downstream, to LESGI-3, rather than speculating on a duplication event of LESGI-3. PAGI-2 was initially described in *P. aeruginosa* clone C isolates (Larbig et al., 2002). This island is divided into two main phage modules, one coding for chromosome-partitioning proteins (soj) and another containing a bacteriophage P4-related integrase (Klockgether et al., 2007). PAGI-2 is present in approximately 40% of *P. aeruginosa* strains (Klockgether et al., 2007).

Annotation of SNPs across the sequenced genomes allowed the identification of a number of moderate or high impact mutations in coding sequences (Table 2). Mutations in virulence genes, like pyoverdine biosynthesis gene *pvdL* and pilin biosynthesis gene *chpA* (*pilL*), are known as hallmarks of adaptation of *P. aeruginosa* strains to the CF airway environment (Winstanley et al., 2016). Siderophore-mediated processes have been linked with virulence regulation of *P. aeruginosa* and its pathogenic potential (Lamont et al., 2002) and non-synonymous mutations were found in *pvdL* in the evolutionary pathway leading to a hypermutable CF DK2 clone (Marvig et al., 2014). However, in a study conducted on 36 CF patients, pyoverdine was undetectable in one-third of sputa positive for *P. aeruginosa* (Martin et al., 2011). The non-synonymous variations of *pvdL* found in the present study have not been characterized to date, and thus their potential role in the defect in the biosynthesis and functionality of the siderophore system cannot be assessed yet. The presence of pili is necessary for the initial colonization and the translocation of type III exotoxins (Hogardt and Heesemann, 2013), but adapted and late CF isolates accumulate mutations in genes associated with pili, and loss of motility is associated with a higher risk of persistent infections (Bragonzi et al., 2009; Bianconi et al., 2015).

Several mutations in genes associated with antibiotic resistance were detected. The outer membrane protein OprD is involved in the resistance against β-lactams and carbapenems (Li et al., 2012). The protein contains 16 β-strands connected by short loops, eight of which are external loops (Huang et al., 1995). The deletion present in the MDR isolates of the population disrupts the protein just before the fourth loop, and it has been reported that the deletion of loops 3 and 4 results in a non-stable expression of the protein (Huang et al., 1995). In *P. aeruginosa* repression or inactivation of OprD or a reduced protein expression contribute to moderate resistance to imipenem (Quale et al., 2006; Rodriguez-Martinez et al., 2009; Poole, 2011), and carbapenem-resistant strains are often defective in expression of OprD (Naenna et al., 2010). Moreover, *P. aeruginosa* PA14 mutants for *oprD* showed an enhanced fitness in a murine model of mucosal colonization and dissemination (Skurnik et al., 2013).

Several variations were found in the genes encoding DNA gyrase subunit B (GyrB) and topoisomerase IV subunit B (ParE), which are both significant contributors to the acquisition of resistance to fluoroquinolones (Lee et al., 2005). Five different variants were detected for *gyrB* in 36 genomes. In particular, amino acid substitutions E468-D and S749-P were found in isolates resistant to fluoroquinolones, whereas the gene encoding ParE showed three different variations within the population, including R30-C and H59-R in the late MDR isolates (Table 3). According to the likelihood ratio test, the null hypothesis of neutrality was rejected for both genes, suggesting that they are under non-neutral selection in the population (Table 2). The amino acid substitution E468-D in GyrB was reported to be associated to a high level of resistance to ciprofloxacin in clinical isolates (Lee et al., 2005; Caballero et al., 2015). This variation is within the quinolone resistance-determining regions (QRDR) of the protein, while the mutations in *parE* are outside the QRDR domain. Nonetheless, non-synonymous variations in *gyrB* and *parE* have a low frequency in clinical isolates, and they are described to have only a complementary role in fluoroquinolone resistance (Lee et al., 2005; Lister et al., 2009).

In a recent study (Bruchmann et al., 2013), most of the *P. aeruginosa* clinical isolates analyzed carried mutations either in *gyrA* or *gyrB* or in both *gyrA* and *parC*. Mutations in *parC* gene, encoding topoisomerase IV subunit A, along with those described above in *gyrB*, are known to confer resistance to fluoroquinolones (Mouneimné et al., 1999; Lee et al., 2005), while clinical isolates with mutations in the QRDR of *gyrA* and *parC* show high levels of fluoroquinolone resistance (Lister et al., 2009). A well-characterized double threonine-serine variation in *gyrA* and *parC* genes responsible for high-level resistance to fluoroquinolones (Agnello et al., 2016; Fuzi et al., 2017) was not found in the this study. However, while *gyrA* carried a G75-A variation only in the seven strains belonging to the ST1864, we identified a yet uncharacterized N170-S substitution in *parC*, present only in the MDR isolates, but this variation is outside the QRDR domain.

The *pmrB* gene encoding the two-component regulatory system signal sensor kinase showed three different variants within the population. PmrB is part of the two-component regulatory system PmrA/PmrB involved in resistance to polymyxins and other cationic antimicrobial peptides in *P. aeruginosa* (Moskowitz et al., 2004, 2012; Barrow and Kwon, 2009). Considering that variations found in this population were not directly associated with an MDR phenotype and that this gene was not found to be under non-neutral selective pressure (likelihood ratio test *p*-value > 0.05), we can assume that the mutations are neutral.

MexG, a component of the efflux pump MexGHI-OpmD, carries an amino acid substitution, P51-S, in nearly all the MDR isolates; mutations in this pump are involved in the resistance to tetracycline and ticarcillin-clavulanate and the export of non-antibiotic compounds, including vanadium (Aendekerk et al., 2002). However, the P51-S variant has not been reported to date. Further investigations are thus needed to assess its putative role in antibiotic resistance.

We observed a significant trend to lose the mucoid phenotype over time in the population, and this phenotype did not correlate with the antibiotic resistance profile of the isolates.

*Pseudomonas aeruginosa* is known to lose mucoid phenotypes in the latest stages of CF lung disease, because the production of alginate represents a high-energy cost for the bacterium (Hogardt and Heesemann, 2010; Sousa and Pereira, 2014). On another hand, conversion to a mucoid phenotype may promote biofilm formation, which is a crucial factor for the persistence of *P. aeruginosa* in CF airways (Sousa and Pereira, 2014) and a sessile lifestyle can efficaciously increase tolerance and resistance to antibiotics (Häussler, 2010; Kostakioti et al., 2013). Biofilm formation assay showed that the isolates grew as sessile community mainly in the intermediate/late stage of infection (Supplementary Table S4). These observations were confirmed by Spearman’s correlation between phenotypes and time. We checked if the differences observed in biofilm formation could be due to mutations in the dual-regulator system PA1226–PA1413 (Heacock-Kang et al., 2018), suppressing the ability of *P. aeruginosa* to form biofilm *in vitro*. No difference in the two genes between poor and good biofilm producers was found in this study.

## Conclusion

The analysis of the genomes of this longitudinal collection of 40 closely related *P. aeruginosa* strains belonging to the same lineage and isolated from a single CF patient over a long-term period provides new insights that increase our knowledge on the adaptation and microevolution of this pathogen inside its human host. The study revealed the emergence of a MDR phenotype over time that could not be comprehensively explained by mutations found in known resistance genes. Further investigations on uncharacterized variations disclosed in this study should help to increase our understanding of the development of MDR phenotypes and the poor outcome of antibiotic therapies in many CF patients.

## Availability of Data and Material

Data analyzed and discussed in this article are included in the article itself or the additional files. Genome sequence data have been deposited at DDBJ/ENA/GenBank under accession numbers MAUO00000000 – MBMR00000000.

## Ethics Statement

Strains were isolated during routine monitoring of the CF patient at the Trentino CF Support Centre, Hospital of Rovereto, Italy. The patient provided informed consent in accordance with the Declaration of Helsinki before sampling of strains and storage of clinical data. Written informed consent for publication of clinical details was obtained from the patient. A copy of the consent form is available for review by the Editor of this journal.

## Author Contributions

OJ and EB jointly designed the study. EB, PG, GD, and MS isolated and identified the samples and interpreted the data. IB, SD’A, and EP prepared the sequencing libraries. IB, SD’A, AE, MB, CD, and OJ analyzed and interpreted the data. IB, AE, and OJ wrote the manuscript. All authors have read and approved the final version of the manuscript.

## Conflict of Interest Statement

The authors declare that the research was conducted in the absence of any commercial or financial relationships that could be construed as a potential conflict of interest.

## Footnotes

1 https://github.com/Sandramses/PAst

2 https://www.rdocumentation.org/packages/ggpubr

3 https://github.com/tseemann/snippy

4 http://202.120.12.133/TAfinder/index.php

5 http://202.120.12.133/STEP/STEP_CDSeasy.php

6 https://www.rdocumentation.org/packages/caper/versions/0.5.2/topics/phylo.d

7 https://pubmlst.org/bigsdb?db=pubmlst_paeruginosa_isolates&page=profiles

8 https://pubmlst.org/
