# Supplementary material for: Persistence and Microevolution of Pseudomonas aeruginosa in the Cystic Fibrosis Lung: A Single-Patient Longitudinal Genomic Study
Source: Front Microbiol. 2019 Jan 11;9:3242. doi: 10.3389/fmicb.2018.03242 (PMC6340092; doi:10.3389/fmicb.2018.03242)
Supplement: Supplementary file 1 [file Image_1.pdf]

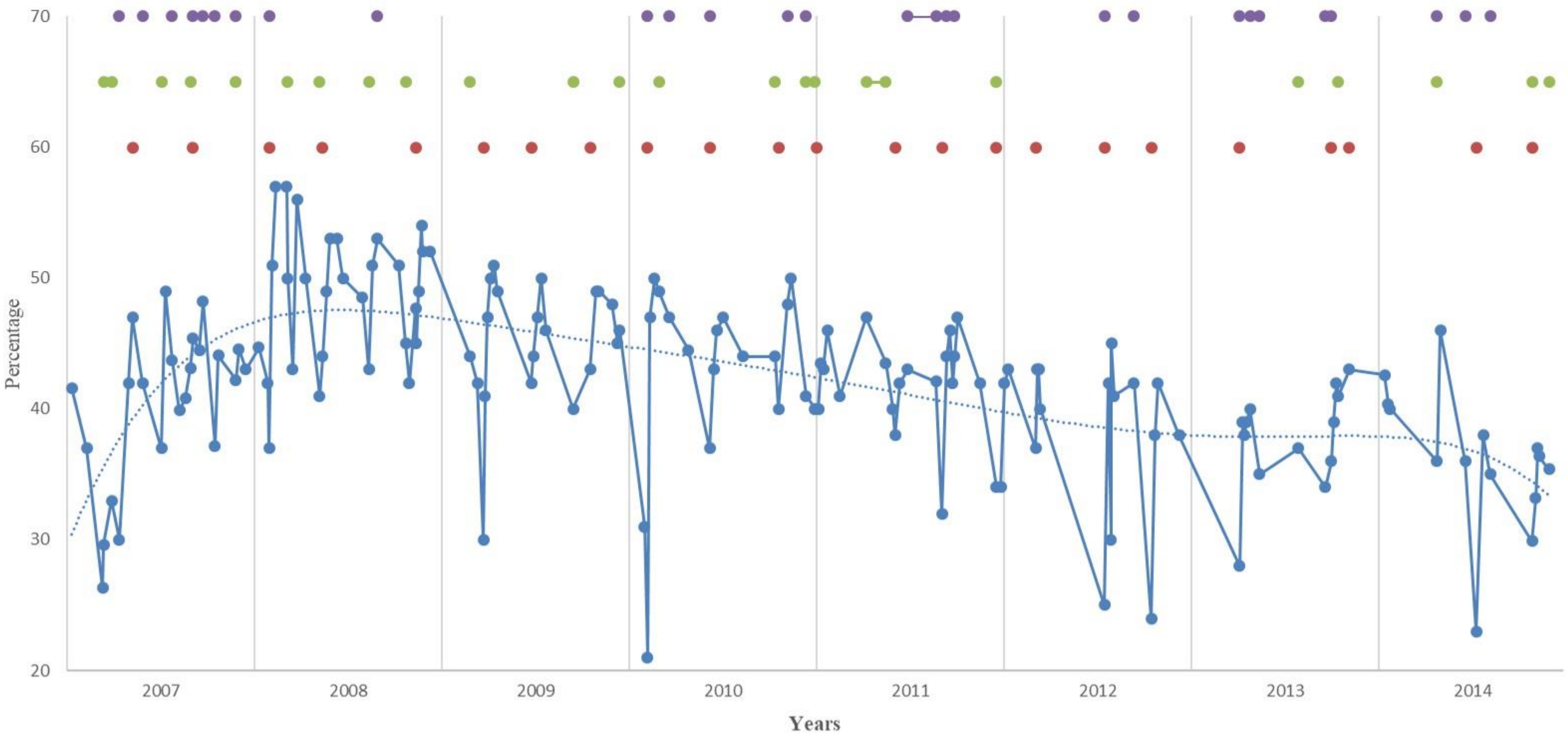

Additional file 1: Figure S1. Time course of FEV1 (Forced Expiratory Volume in the 1st second), antibiotic therapies and strain isolation. Intravenously administered antibiotics are shown in red; orally administered drugs are in green, isolation of bacterial strains from sputum samples is shown in purple. FEV1 values (percentages) and the trendline of FEV1 time course are shown.
