# Supplementary material for: Persistence and Microevolution of Pseudomonas aeruginosa in the Cystic Fibrosis Lung: A Single-Patient Longitudinal Genomic Study
Source: Front Microbiol. 2019 Jan 11;9:3242. doi: 10.3389/fmicb.2018.03242 (PMC6340092; doi:10.3389/fmicb.2018.03242)

Additional file 4: Figure S2. Analysis of recombination in the population performed with ClonalFrameML. The input tree topology was that produced by BEAST (Fig. 2); branch length is corrected accounting the contribution of putative recombinant characters. The likelihood of recombination for each site along the concatenated core genomes (x-axis of the right panel) is shown on the heatmap, with colour codes as follows: light blue, low likelihood; yellow to orange, increasing likelihood; dark blue, highest likelihood.

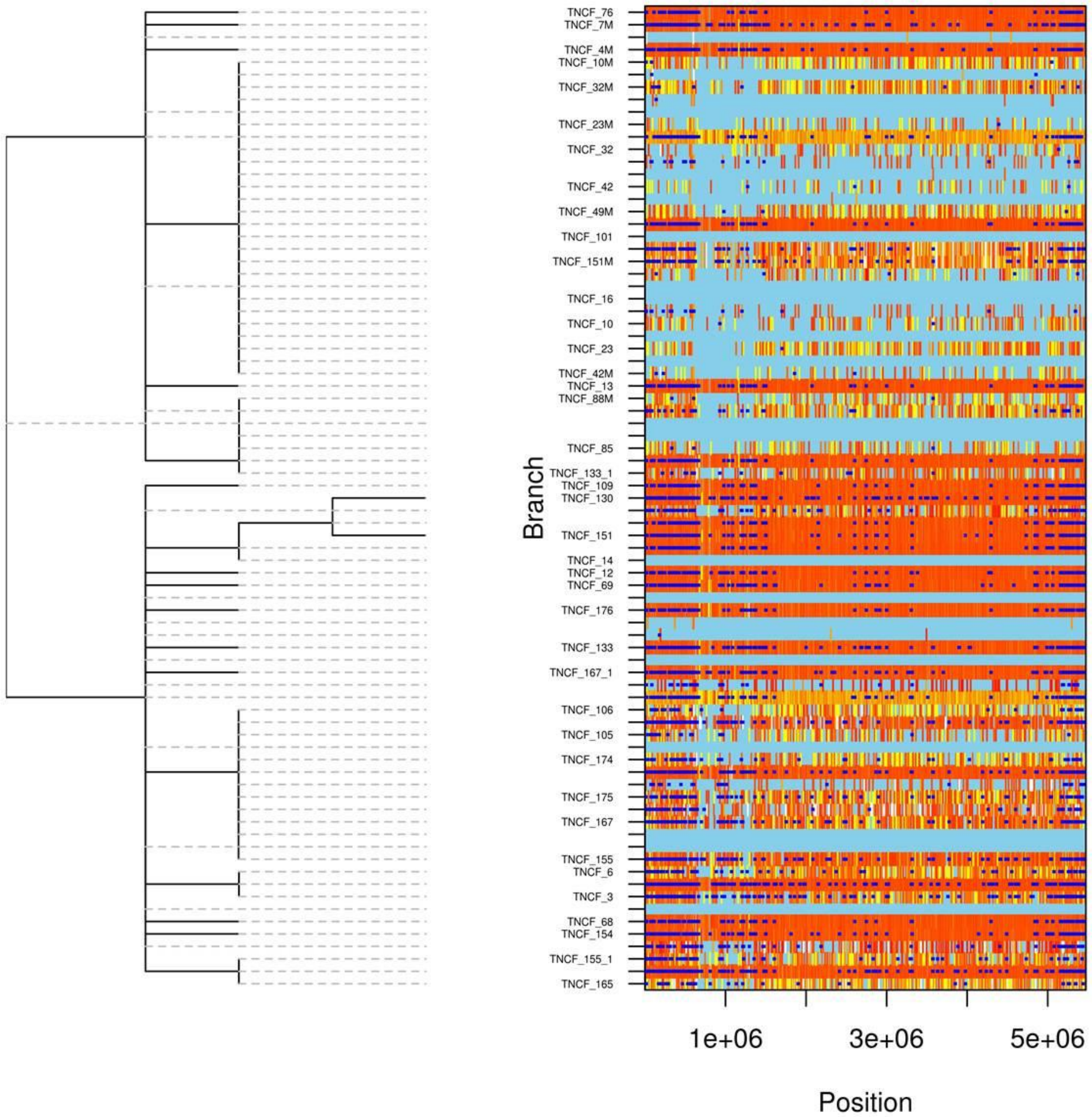

Supplement: Supplementary file 2 [file Image_2.pdf]
