# Supplementary material for: Persistence and Microevolution of Pseudomonas aeruginosa in the Cystic Fibrosis Lung: A Single-Patient Longitudinal Genomic Study
Source: Front Microbiol. 2019 Jan 11;9:3242. doi: 10.3389/fmicb.2018.03242 (PMC6340092; doi:10.3389/fmicb.2018.03242)

Additional file 5: Figure S3. Phylogenetic tree based on the distribution of accessory genes in the *P. aeruginosa* population. The analysis was performed using the hclust() function from the base R package; core genes have been removed from the analysis to improve the resolution of the picture.

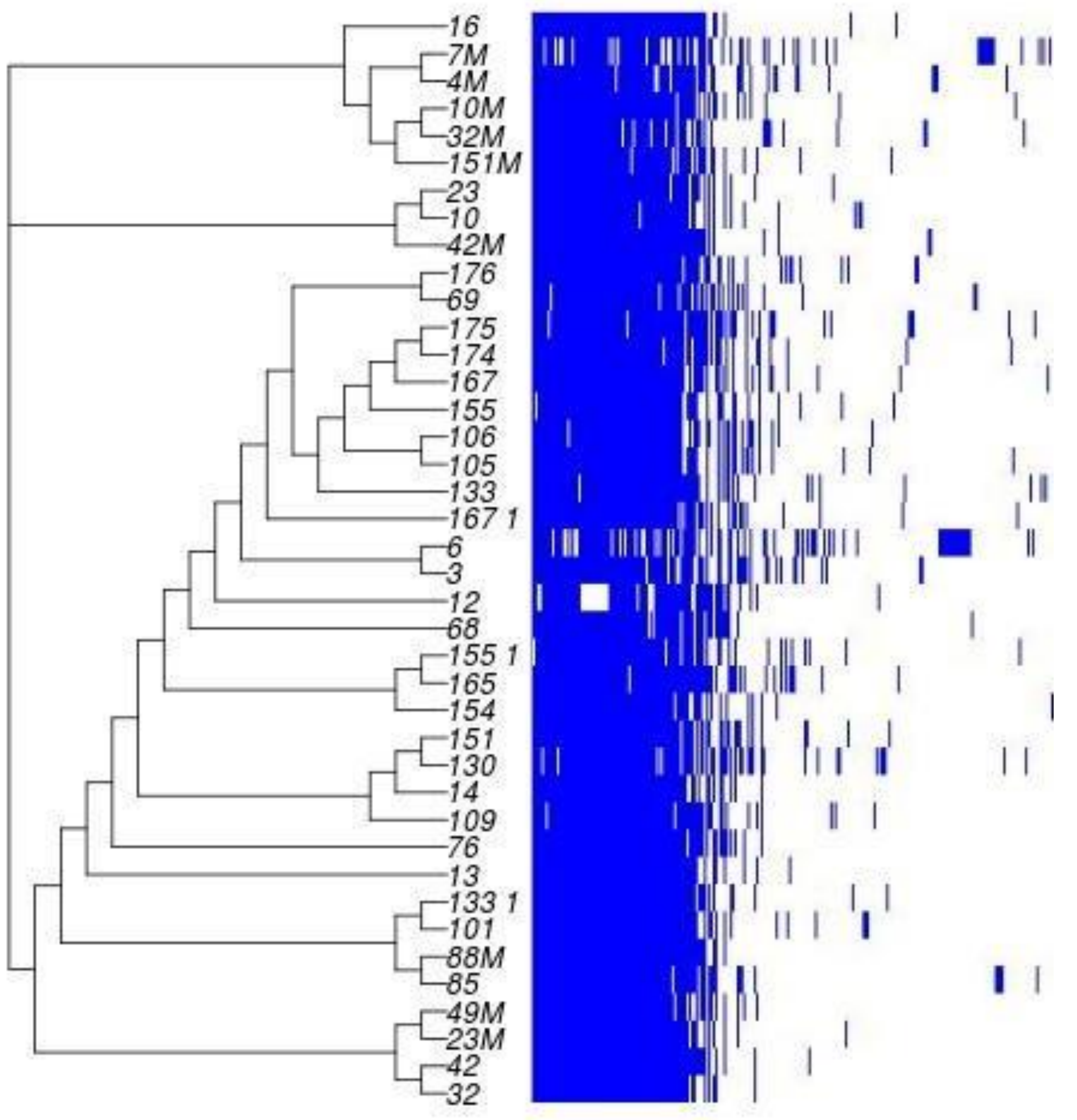

Supplement: Supplementary file 3 [file Image_3.pdf]
