# Supplementary material for: Persistence and Microevolution of Pseudomonas aeruginosa in the Cystic Fibrosis Lung: A Single-Patient Longitudinal Genomic Study
Source: Front Microbiol. 2019 Jan 11;9:3242. doi: 10.3389/fmicb.2018.03242 (PMC6340092; doi:10.3389/fmicb.2018.03242)

**Additional file 8: Figure S6. Heatmap representation of the distance matrix between pairs of isolates based on core genome variants with moderate impact.** The graph was obtained using the R-function heatmap() with all default parameters.

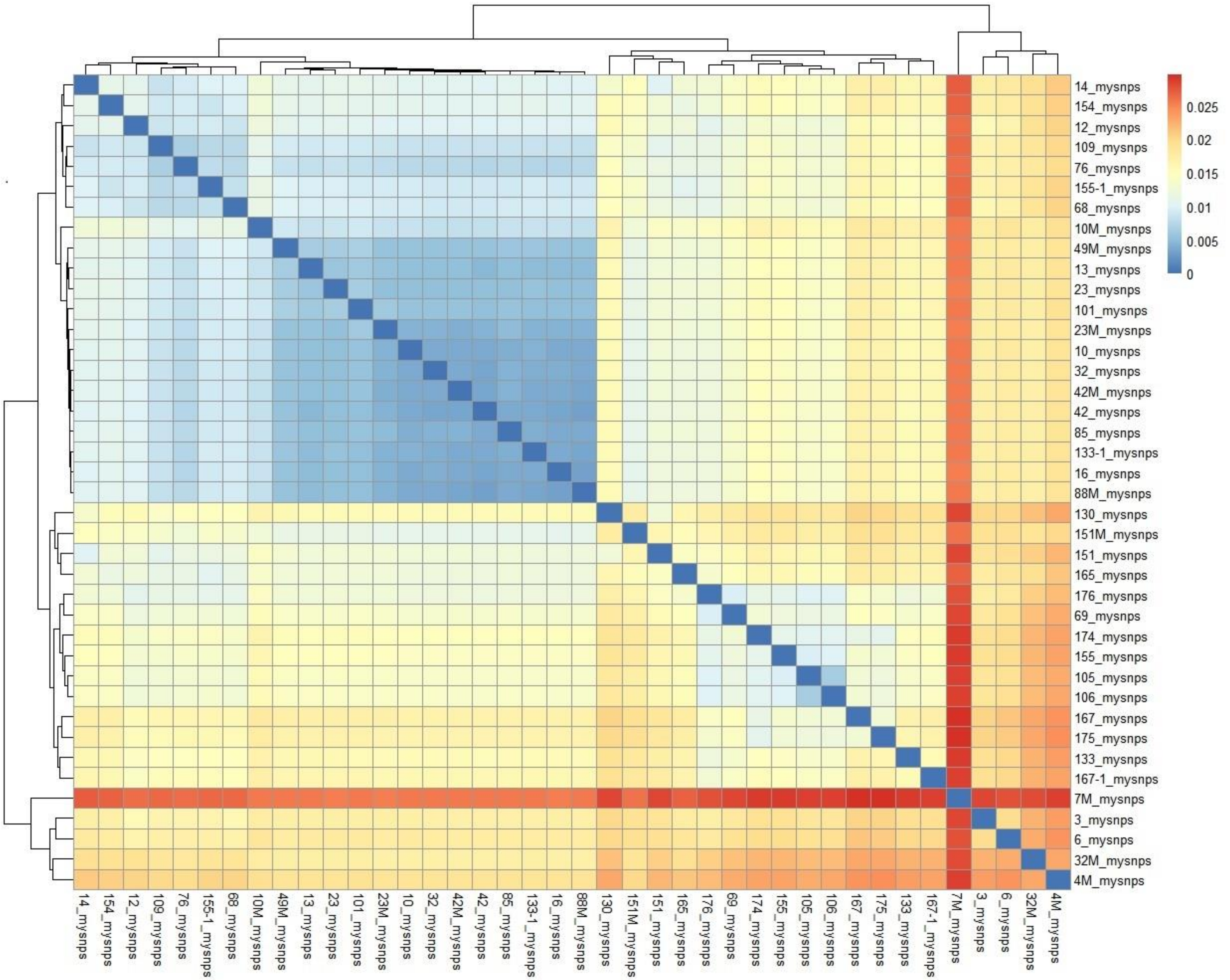

Supplement: Supplementary file 6 [file Image_6.pdf]
