# Supplementary material for: Persistence and Microevolution of Pseudomonas aeruginosa in the Cystic Fibrosis Lung: A Single-Patient Longitudinal Genomic Study
Source: Front Microbiol. 2019 Jan 11;9:3242. doi: 10.3389/fmicb.2018.03242 (PMC6340092; doi:10.3389/fmicb.2018.03242)
Supplement: Supplementary file 7 [file Image_7.pdf]

Additional file 9: Figure S7. Increase over time of multidrug-resistant isolates. Isolates are ordered according to sampling time. Resistant, intermediate, and susceptible isolates are highlighted in red, yellow and green, respectively.

Isolate	Aminoglycosides		ß-lactams		Carbapenem	Ceftazidime	Cefepime	Fluoroquinolones		Phosphonic	Polymyxin	Penicillin/ß-lactam
	Amikacin	Gentamicin	Imipenem	Meropenem	Doripenem			Ciprofloxacin	Levofloxacin	Fosfo.G6fos	Colistin	Piper/Tazo
TNCF_3	[illegible]	[illegible]	[illegible]	[illegible]	[illegible]	[illegible]	[illegible]	[illegible]	[illegible]	[illegible]	1	[illegible]
TNCF_4M	8	4	1	≤0.12	≤0.5	0.25	2	≤0.25	1	16	≤0.5	≤2/4
TNCF_6	[illegible]	[illegible]	[illegible]	[illegible]	[illegible]	[illegible]	[illegible]	[illegible]	[illegible]	[illegible]	≤0.5	[illegible]
TNCF_7M	8	2	1	≤0.12	0.5	0.5	4	0.5	1	32	1	≤2/4
TNCF_10	≤4	2	≤1	≤0.12	≤0.5	1	4	≤0.25	≤1	32	1	≤2/4
TNCF_10M	≤4	2	≤1	≤0.12	≤0.5	0.25	4	0.25	≤1	≤16	≤0.5	≤2/4
TNCF_12	[illegible]	[illegible]	[illegible]	[illegible]	[illegible]	[illegible]	[illegible]	[illegible]	[illegible]	[illegible]	≤0.5	[illegible]
TNCF_13	8	4	2	≤0.12	≤0.5	2	[illegible]	0.5	≤1	[illegible]	1	≤2/4
TNCF_14	[illegible]	[illegible]	[illegible]	8	[illegible]	[illegible]	[illegible]	[illegible]	[illegible]	[illegible]	≤0.5	[illegible]
TNCF_16	[illegible]	4	2	≤0.12	≤0.5	1	4	≤0.25	≤1	32	1	≤2/4
TNCF_23	≤4	2	≤1	≤0.12	≤0.5	2	[illegible]	0.25	≤1	32	2	≤2/4
TNCF_23M	≤4	2	≤1	≤0.12	2	1	[illegible]	0.5	≤1	32	1	≤2/4
TNCF_32	8	4	≤1	≤0.12	≤0.5	1	[illegible]	0.5	2	[illegible]	1	≤2/4
TNCF_32M	≤4	2	≤1	≤0.12	≤0.5	1	[illegible]	0.25	1	32	1	≤2/4
TNCF_42	≤4	2	≤1	≤0.12	≤0.5	1	[illegible]	0.5	≤1	32	1	≤2/4
TNCF_42M	4	1	1	≤0.12	≤0.5	0.25	4	≤0.25	1	16	1	≤2/4
TNCF_49M	≤4	2	≤1	≤0.12	≤0.5	2	[illegible]	0.25	≤1	32	1	≤2/4
TNCF_68	[illegible]	[illegible]	[illegible]	[illegible]	[illegible]	[illegible]	[illegible]	[illegible]	[illegible]	[illegible]	1	[illegible]
TNCF_69	[illegible]	[illegible]	[illegible]	[illegible]	[illegible]	[illegible]	[illegible]	[illegible]	[illegible]	[illegible]	≤0.5	[illegible]
TNCF_76	[illegible]	[illegible]	>16	16	[illegible]	[illegible]	[illegible]	[illegible]	[illegible]	[illegible]	1	[illegible]
TNCF_85	16	4	2	0.25	≤0.5	2	[illegible]	[illegible]	[illegible]	[illegible]	≤0.5	43/98
TNCF_88M	8	2	4	0.25	≤0.5	2	2	[illegible]	[illegible]	[illegible]	≤0.5	≤2/4
TNCF_101	[illegible]	[illegible]	[illegible]	[illegible]	[illegible]	[illegible]	[illegible]	[illegible]	[illegible]	[illegible]	≤0.5	[illegible]
TNCF_105	[illegible]	[illegible]	[illegible]	[illegible]	[illegible]	[illegible]	[illegible]	[illegible]	[illegible]	[illegible]	≤0.5	[illegible]
TNCF_106	[illegible]	[illegible]	[illegible]	[illegible]	[illegible]	[illegible]	[illegible]	1	2	[illegible]	≤0.5	[illegible]
TNCF_109	[illegible]	[illegible]	[illegible]	[illegible]	[illegible]	[illegible]	[illegible]	[illegible]	[illegible]	[illegible]	≤0.5	[illegible]
TNCF_130	[illegible]	[illegible]	[illegible]	8	[illegible]	[illegible]	[illegible]	[illegible]	[illegible]	[illegible]	≤0.5	[illegible]
TNCF_133	[illegible]	[illegible]	[illegible]	[illegible]	[illegible]	[illegible]	[illegible]	[illegible]	[illegible]	[illegible]	≤0.5	[illegible]
TNCF_133_1	[illegible]	[illegible]	[illegible]	2	2	[illegible]	[illegible]	[illegible]	[illegible]	[illegible]	≤0.5	[illegible]
TNCF_151	[illegible]	[illegible]	[illegible]	[illegible]	[illegible]	[illegible]	[illegible]	[illegible]	[illegible]	[illegible]	1	[illegible]
TNCF_151M	[illegible]	4	≤1	≤0.12	≤0.5	0.25	2	0.25	≤1	≤16	≤0.5	≤2/4
TNCF_154	[illegible]	[illegible]	[illegible]	[illegible]	[illegible]	[illegible]	[illegible]	[illegible]	[illegible]	[illegible]	1	[illegible]
TNCF_155	[illegible]	[illegible]	[illegible]	[illegible]	[illegible]	[illegible]	[illegible]	[illegible]	[illegible]	[illegible]	≤0.5	[illegible]
TNCF_155_1	[illegible]	[illegible]	[illegible]	[illegible]	[illegible]	[illegible]	[illegible]	[illegible]	[illegible]	[illegible]	1	[illegible]
TNCF_165	[illegible]	[illegible]	[illegible]	[illegible]	[illegible]	[illegible]	[illegible]	[illegible]	[illegible]	[illegible]	1	[illegible]
TNCF_167	[illegible]	[illegible]	[illegible]	[illegible]	[illegible]	[illegible]	[illegible]	[illegible]	[illegible]	[illegible]	≤0.5	[illegible]
TNCF_167_1	[illegible]	[illegible]	[illegible]	[illegible]	[illegible]	[illegible]	[illegible]	[illegible]	[illegible]	[illegible]	1	[illegible]
TNCF_174	[illegible]	[illegible]	[illegible]	[illegible]	[illegible]	[illegible]	[illegible]	[illegible]	[illegible]	[illegible]	≤0.5	[illegible]
TNCF_175	[illegible]	[illegible]	[illegible]	[illegible]	[illegible]	[illegible]	[illegible]	1	[illegible]	32	≤0.5	[illegible]
TNCF_176	[illegible]	[illegible]	[illegible]	[illegible]	[illegible]	[illegible]	[illegible]	[illegible]	[illegible]	[illegible]	1	[illegible]
