# Supplementary material for: Persistence and Microevolution of Pseudomonas aeruginosa in the Cystic Fibrosis Lung: A Single-Patient Longitudinal Genomic Study
Source: Front Microbiol. 2019 Jan 11;9:3242. doi: 10.3389/fmicb.2018.03242 (PMC6340092; doi:10.3389/fmicb.2018.03242)
Supplement: Supplementary file 8 [file Image_8.pdf]

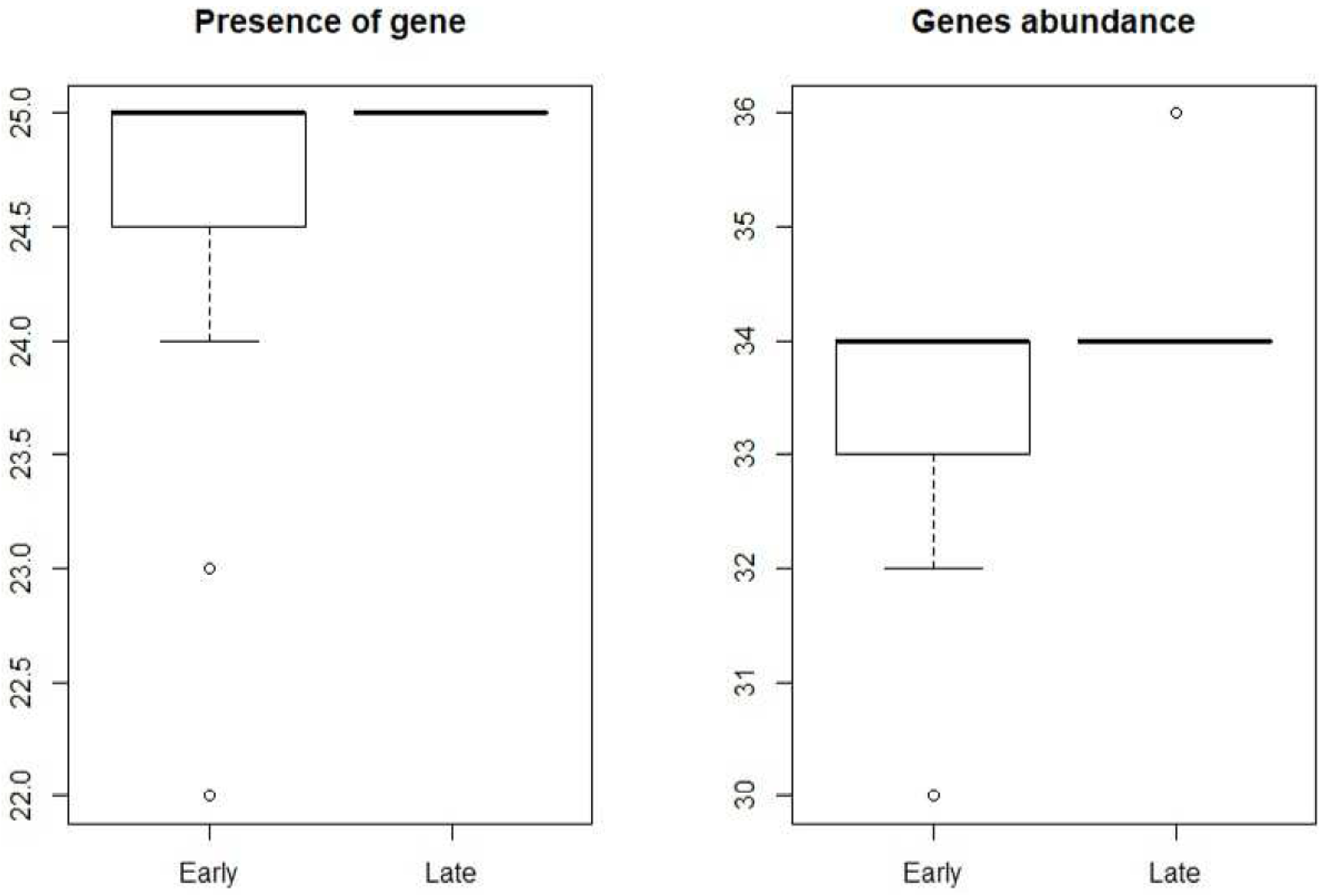

Additional file 10: Figure S8: Boxplots of TA genes.
Boxplots resuming the number of TA genes (left) and their total abundance (right) in the genomes from early and late strains.
