# Supplementary material for: Persistence and Microevolution of Pseudomonas aeruginosa in the Cystic Fibrosis Lung: A Single-Patient Longitudinal Genomic Study
Source: Front Microbiol. 2019 Jan 11;9:3242. doi: 10.3389/fmicb.2018.03242 (PMC6340092; doi:10.3389/fmicb.2018.03242)

Additional file 14: Figure S9: Piechart resuming SNPs distribution.
Distribution of high impact SNPs found in the population according to COG categories.

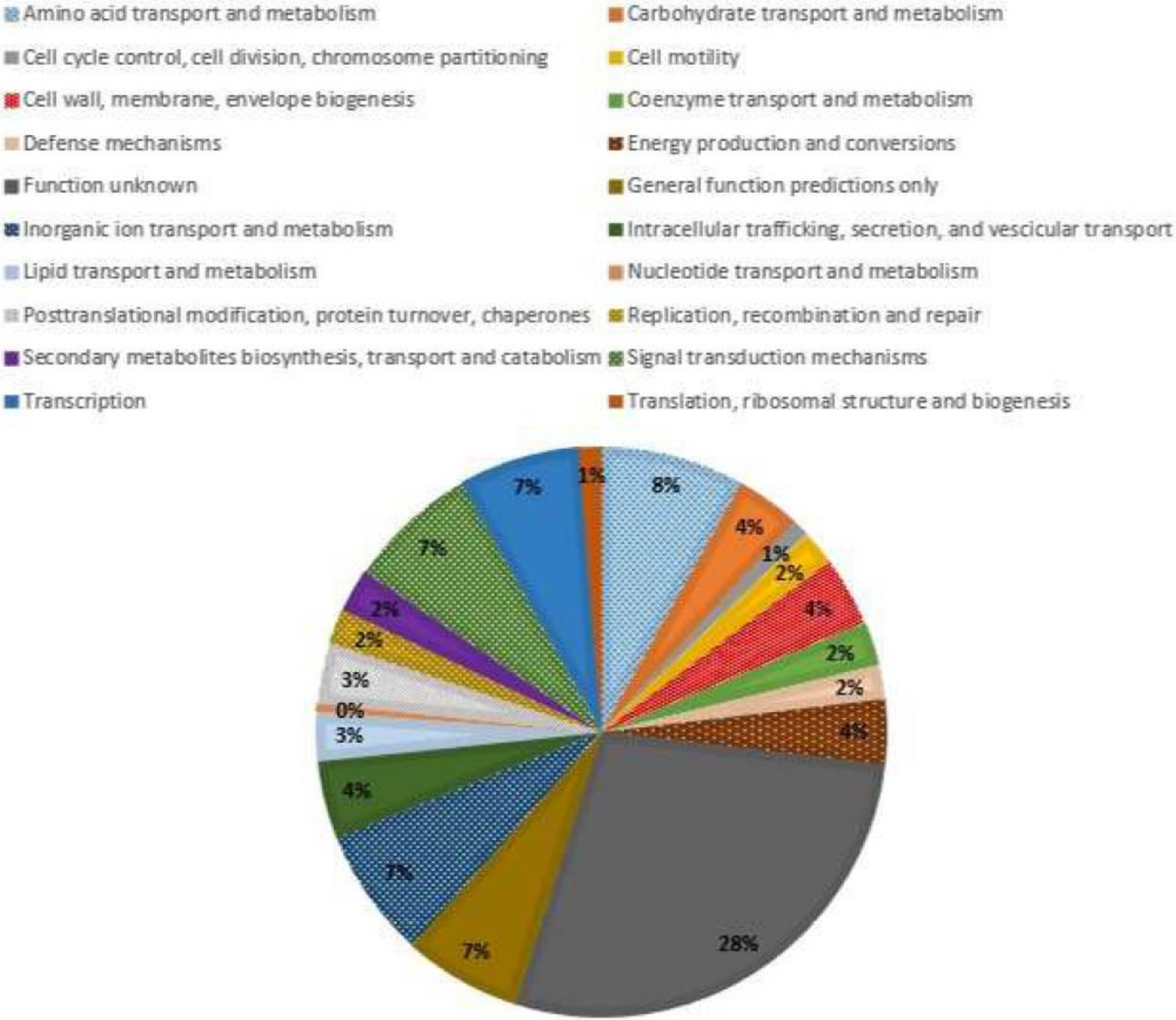

Supplement: Supplementary file 9 [file Image_9.pdf]
