# Supplementary material for: Persistence and Microevolution of Pseudomonas aeruginosa in the Cystic Fibrosis Lung: A Single-Patient Longitudinal Genomic Study
Source: Front Microbiol. 2019 Jan 11;9:3242. doi: 10.3389/fmicb.2018.03242 (PMC6340092; doi:10.3389/fmicb.2018.03242)
Supplement: Supplementary file 10 [file Table_1.pdf]

Additional file 2: Table S1. Antibiotics therapies administered to the patent during the course of this study.

Date	FEV1 %	Antibiotics e.v.	Antibiotics per os
11/01/2007	41.60		
09/02/2007	37.00		
12/03/2007	26.30		
14/03/2007	29.65		ciprofloxacin
30/03/2007	33.00		cefalexin
13/04/2007	30.00		
02/05/2007	42.00		
10/05/2007	47.00	ceftazidime, tobramycin, targocid	
29/05/2007	42.00		
05/07/2007	37.00		ciprofloxacin
13/07/2007	49.00		
26/07/2007	43.70		
09/08/2007	39.90		
21/08/2007	40.80		
31/08/2007	43.10		ciprofloxacin
04/09/2007	45.40	targocid, ceftazidime	
17/09/2007	44.50		
24/09/2007	48.20		
17/10/2007	37.20		
24/10/2007	44.10		
27/11/2007	42.20		azithromycin
03/12/2007	44.60		
17/12/2007	43.00		
11/01/2008	44.70		
28/01/2008	42.00		
31/01/2008	37.00	teicoplanin, tobramycin, ceftazidime	
06/02/2008	51.00		
13/02/2008	57.00		
05/03/2008	57.00		
07/03/2008	50.00		ciprofloxacin
17/03/2008	43.00		
26/03/2008	56.00		
11/04/2008	50.00		
09/05/2008	41.00		ciprofloxacin
14/05/2008	44.00	teicoplanin, tobramycin, ceftazidime	
22/05/2008	49.00		
29/05/2008	53.00		
12/06/2008	53.00		
23/06/2008	50.00		
31/07/2008	48.50		
13/08/2008	43.00		cefalexin
20/08/2008	51.00		

29/08/2008	53.00		
10/10/2008	51.00		
24/10/2008	45.00		ciprofloxacin
31/10/2008	42.00		
12/11/2008	47.70		
13/11/2008	45.00	teicoplanin, tobramycin, ceftazidime	
18/11/2008	49.00		
24/11/2008	54.00		
26/11/2008	52.00		
10/12/2008	52.00		
26/02/2009	44.00		ciprofloxacin
13/03/2009	42.00		
25/03/2009	30.00	teicoplanin, tobramycin, ceftazidime	
27/03/2009	41.00		
01/04/2009	47.00		
07/04/2009	50.00		
14/04/2009	51.00		
21/04/2009	49.00		
26/06/2009	42.00	teicoplanin, tobramycin, ceftazidime	
30/06/2009	44.00		
08/07/2009	47.00		
15/07/2009	50.00		
24/07/2009	46.00		
16/09/2009	40.00		ciprofloxacin
19/10/2009	43.00	piperacillin, tazobactam, teicoplanin	
30/10/2009	49.00		
04/11/2009	49.00		
01/12/2009	48.00		
11/12/2009	45.00		
15/12/2009	46.00		ciprofloxacin
02/02/2010	31.00		
08/02/2010	21.00	piperacillin, tazobactam, teicoplanin	
12/02/2010	47.00		
21/02/2010	50.00		
02/03/2010	49.00		ciprofloxacin
22/03/2010	47.00		
16/04/2010			
28/04/2010	44.50		
09/06/2010	37.00	piperacillin, tazobactam, teicoplanin	
17/06/2010	43.00		
23/06/2010	46.00		
05/07/2010	47.00		
13/08/2010	44.00		

15/10/2010	44.00		ciprofloxacin
21/10/2010	40.00	tazocin, targocid	
08/11/2010	48.00		
15/11/2010	50.00		
14/12/2010	41.00		doxycycline
31/12/2010	40.00		doxycycline
03/01/2011	40.00	piperacillin, tazobactam, teicoplanin	
07/01/2011	40.00		
12/01/2011	43.50		
17/01/2011	43.00		
25/01/2011	46.00		
18/02/2011	41.00		
11/04/2011	47.00		doxycycline
19/05/2011	43.50		doxycycline
01/06/2011	40.00		
06/06/2011	38.00	tazocin, targocid	
15/06/2011	42.00		
30/06/2011	43.00		
25/08/2011	42.10		
06/09/2011	32.00	piperacillin, ceftazidime, teicoplanin	
14/09/2011	44.00		
21/09/2011	46.00		
26/09/2011	42.00		
30/09/2011	44.00		
06/10/2011	47.00		
18/11/2011	42.00		
21/12/2011	34.00	tazocin	linezolid
29/12/2011	34.00		
05/01/2012	42.00		
13/01/2012	43.00		
06/03/2012	37.00	piperacillin, tazobactam, teicoplanin	
09/03/2012	43.00		
12/03/2012	43.00		
15/03/2012	40.00		
02/05/2012			
19/07/2012	25.00	piperacillin, tazobactam, teicoplanin	
26/07/2012	42.00		
31/07/2012	2.65		
01/08/2012	45.00		
06/08/2012	41.00		
14/09/2012	42.00		
18/10/2012	24.00	tazocin, targocid	
25/10/2012	38.00		

31/10/2012	42.00		
12/12/2012	38.00		
08/04/2013	28.00	piperacillin, tazobactam, teicoplanin	
14/04/2013	39.00		
17/04/2013	38.00		
22/04/2013	39.00		
29/04/2013	40.00		
17/05/2013	35.00		
01/08/2013	37.00		linezolid
23/09/2013	34.00		
04/10/2013	36.00	piperacillin, tazobactam, teicoplanin	
09/10/2013	39.00		
14/10/2013	42.00		
18/10/2013	41.00		linezolid
08/11/2013	43.00		
10/01/2014		teicoplanin, meropenem, amikacin	
16/01/2014	42.60		
23/01/2014	40.40		
27/01/2014	40.00		
28/04/2014	36.00		linezolid
05/05/2014	46.00		
23/06/2014	36.00		
14/07/2014	23.00	amikacin, targocid, tazocin	
28/07/2014	38.00		
11/08/2014	35.00		
31/10/2014	29.90	meropenem, amikacin	linezolid
07/11/2014	33.20		
11/11/2014	37.00		
14/11/2014	36.40		
03/12/2014	35.40		linezolid
