# Supplementary material for: Persistence and Microevolution of Pseudomonas aeruginosa in the Cystic Fibrosis Lung: A Single-Patient Longitudinal Genomic Study
Source: Front Microbiol. 2019 Jan 11;9:3242. doi: 10.3389/fmicb.2018.03242 (PMC6340092; doi:10.3389/fmicb.2018.03242)
Supplement: Supplementary file 11 [file Table_2.pdf]

Additional file 3: Table S2. Genome assembly statistics and shared total, high and moderate impact SNPs of the 40 *P. aeruginosa* isolates.

Genome accession number	Isolate name	Year of isolation	Sequence type	Number of contigs	Genome size (Kbp)	N50 (Kbp)	%GC	Total SNPs	High impact variants	Moderate impact variants
MAUO00000000	TNCF_3	2007	390	139	6.636	92	66.28	23926	1	258
MAUP00000000	TNCF_4M	2007	390	161	6.630	78	66.29	18451	0	186
MAUQ00000000	TNCF_6	2007	390	356	6.618	31	66.28	16917	0	243
MAUR00000000	TNCF_7M	2007	390	259	6.623	47	66.28	12983	0	132
MAUS00000000	TNCF_10	2007	390	101	6.643	143	66.28	28588	0	231
MAUT00000000	TNCF_10M	2007	390	107	6.633	111	66.29	27377	0	227
MAZG00000000	TNCF_12	2007	390	102	6.545	177	66.36	27551	0	280
MAZI00000000	TNCF_13	2007	390	75	6.637	195	66.27	28381	0	231
MAZH00000000	TNCF_14	2007	390	89	6.633	158	66.28	28893	1	297
MAKL00000000	TNCF_16	2007	1864	59	6.638	269	66.28	28697	0	230
MAZJ00000000	TNCF_23	2007	390	71	6.635	228	66.28	28430	0	232
MAZK00000000	TNCF_23M	2007	390	64	6.636	228	66.28	28668	0	231
MAKM00000000	TNCF_32	2007	390	67	6.639	229	66.28	28668	0	230
MAZL00000000	TNCF_32M	2007	390	138	6.627	93	66.28	21302	0	194
MAZM00000000	TNCF_42	2008	390	70	6.639	228	66.28	28682	0	230
MAZN00000000	TNCF_42M	2008	390	71	6.640	228	66.28	28691	0	230
MAZO00000000	TNCF_49M	2008	390	76	6.635	177	66.29	28187	0	230
MAZP00000000	TNCF_68	2010	390	82	6.633	162	66.28	28277	0	269
MAZQ00000000	TNCF_69	2010	1863	88	6.639	150	66.28	29398	0	344
MAZR00000000	TNCF_76	2010	390	61	6.634	281	66.28	28793	0	251
MAZS00000000	TNCF_85	2010	1864	101	6.644	124	66.29	28605	0	231
MAZT00000000	TNCF_88M	2010	1864	65	6.636	229	66.28	28678	0	231
MAZU00000000	TNCF_101	2011	1864	142	6.653	92	66.28	28541	0	229
MAZV00000000	TNCF_105	2011	390	92	6.644	191	66.28	29525	0	338
MAZW00000000	TNCF_106	2011	390	77	6.634	205	66.28	29346	0	336
MAZX00000000	TNCF_109	2011	390	69	6.634	205	66.28	28808	0	264
MAZD00000000	TNCF_130	2012	390	157	6.625	76	66.28	24731	1	312
MAZF00000000	TNCF_133	2012	390	82	6.637	154	66.29	29356	2	374
MAZE00000000	TNCF_133_1	2012	1864	87	6.641	269	66.28	28687	0	231
MAKK00000000	TNCF_151	2013	390	53	6.629	378	66.28	29377	1	340
MBMI00000000	TNCF_151M	2013	1864	103	6.636	143	66.28	26177	0	221
MBMJ00000000	TNCF_154	2013	390	86	6.635	177	66.28	27323	0	254
MBMK00000000	TNCF_155	2013	390	62	6.634	339	66.28	29744	0	353
MBML00000000	TNCF_155_1	2013	1923	71	6.635	221	66.28	28689	0	255
MBMM00000000	TNCF_165	2013	1923	119	6.634	135	66.28	26654	0	260
MBMN00000000	TNCF_167	2013	390	73	6.634	191	66.27	29024	2	392
MBMO00000000	TNCF_167_1	2013	390	91	6.628	143	66.28	27629	0	335
MBMP00000000	TNCF_174	2014	390	111	6.645	143	66.29	29317	1	353
MBMQ00000000	TNCF_175	2014	390	118	6.642	124	66.28	29582	1	393
MBMR00000000	TNCF_176	2014	1923	61	6.637	354	66.28	29299	0	304
