# Supplementary material for: Persistence and Microevolution of Pseudomonas aeruginosa in the Cystic Fibrosis Lung: A Single-Patient Longitudinal Genomic Study
Source: Front Microbiol. 2019 Jan 11;9:3242. doi: 10.3389/fmicb.2018.03242 (PMC6340092; doi:10.3389/fmicb.2018.03242)
Supplement: Supplementary file 12 [file Table_3.pdf]

Additional file 11: Table S3. Non-neutral evolution test by LTR, and Tajima's D values for each gene selected for the analysis. See the main text for details about the non-neutrality test comparing models of evolution, Tajima's D was calculated to have furhter evidence of non-neutral evolution.

gene name	omega (M1a)	omega (M2a)	LogL H0 (M1a)	LogL H1	Likelihood Ratio Test (2*(l1-l0)) with 2 df (>5,991)	under selective pressure	omega (M7)	omega (M8)	LogL H0 (M7)	LogL H1	Likelihood Ratio Test (2*(l1-l0)) with 2 df	under selective pressure	Tajima's D
aruF		0,402	0,402	-1.291,9725	-1.291,9740	-0,0030	no	0,402	0,402	-1.291,9725	-1.291,9728	-0,0006	no
cbrA		1	1,12	-883,3932	-883,3882	0,0101	no	1	1,12	-883,3932	-883,3882	0,0101	no
chpA	0,77	1,417	-10.486,4352	-10.474,7069	23,4566	yes	1	1,417	-10.486,6790	-10.474,7078	23,9424	yes	-1,93
ftsJ		1	1,12	-883,3932	-883,3882	0,0101	no	1	1,12	-883,3932	-883,3882	0,0101	no
gidA	0,501	2,241	-2.696,9706	-2.688,2459	17,4495	yes	0,5	2,241	-2.696,9707	-2.688,2462	17,4490	yes	-1,65
glnE	1	1	-4.164,6365	-4.164,6365	0,0000	no	1	1,318	-4.164,6365	-4.164,5473	0,1785	no	-1,98
gltB	0,618	2,179	-6.159,6129	-6.149,4395	20,3468	yes	0,4	2,986	-6.168,9490	-6.150,0270	37,8441	yes	-1,36
gyrB	1	13,00	-3.380,5391	-3.370,0833	20,9115	yes	1	13,49	-3.380,5392	-3.370,9076	19,2631	yes	-0,06
nagE	1	38,787	-2.484,3199	-2.473,3311	21,9775	yes	1	38,78	-2.484,3188	-2.473,3298	21,9780	yes	-2,26
ParE	1	24	-2.655,0258	-2.645,9685	18,1145	yes	1	24,4	-2.655,0265	-2.645,9685	18,1159	yes	-0,66
PmrB	1	2,16	-2.005,4163	-2.005,1474	0,5378	no	1	2,1611	-2.005,4163	-2.005,1543	0,5240	no	-0,8
pvdL	\	\	\	\	\	\	\	\	\	\	\	\	N/A
*15595416 **	0,486	0,486	-2.140,5747	-2.140,5747	0,0000	no	0,485	0,486	-2.140,5749	-2.140,5749	0,0000	no	-2,33
*15595440 **	1	1,573	-942,6485	-942,5663	0,1642	no	1	1,573	-942,6488	-942,5659	0,1658	no	-1,87
*15595766 **		1	80,768	-655,5545	-654,6594	1,7902	no	1	998	-655,5546	-654,6460	1,8172	no
*15596248 **	1	3,5	-1.940,2519	-1.937,2854	5,9331	no	1	3,5	-1.940,2519	-1.937,2854	5,9331	no	-2,03
*15596746 **	0,522	2,042	-3.438,3693	-3.427,6205	21,4976	yes	0,5	2,034	-3.438,3685	-3.427,6870	21,3631	yes	-1,61
*15597414 **	1	999	-1.542,2655	-1.540,7609	3,0093	no	1	998	-1.542,2655	-1.540,7609	3,0093	no	-1,64
*15597654 **	0,2320	2,19	-1.560,9988	-1.544,3529	33,2918	yes	0,3	2,191	-1.561,0650	-1.544,3530	33,4240	yes	-1,4
*15599121 **	0,5361	1,7982	-1.683,4464	-1.676,3017	14,2893	yes	1	1,7355	-1.683,8311	-1.676,3156	15,0312	yes	-1,7
