# Supplementary material for: Persistence and Microevolution of Pseudomonas aeruginosa in the Cystic Fibrosis Lung: A Single-Patient Longitudinal Genomic Study
Source: Front Microbiol. 2019 Jan 11;9:3242. doi: 10.3389/fmicb.2018.03242 (PMC6340092; doi:10.3389/fmicb.2018.03242)
Supplement: Supplementary file 13 [file Table_4.pdf]

Additional file 12: Table S4. Phenotypic assays. Qualitative assessment of mucoid phenotype and biofilm formation assays are reported.

Isolate	Mucoid phenotype	Biofilm production (OD 695/600)	
		Mean	SD
TNCF_3	NO	0.2538	0.2767
TNCF_4M	YES	0.0266	0.0392
TNCF_6	NO	0.1719	0.1415
TNCF_7M	YES	0.0185	0.0163
TNCF_10	NO	0.0059	0.0098
TNCF_10M	YES	0.0132	0.0187
TNCF_12	NO	0.0563	0.0381
TNCF_13	YES	0.0049	0.0075
TNCF_14	NO	1.1492	1.4577
TNCF_16	YES	0.0232	0.0246
TNCF_23	NO	0.0056	0.0092
TNCF_23M	YES	0.0046	0.0074
TNCF_32	NO	0.0294	0.0641
TNCF_32M	YES	0.004	0.0072
TNCF_42	NO	0.0032	0.0051
TNCF_42M	YES	0.0035	0.0042
TNCF_49M	YES	0.0136	0.0236
TNCF_68	NO	0.1404	0.086
TNCF_69	NO	0.3606	0.2108
TNCF_76	NO	53.8531	16.8565
TNCF_85	YES	0.0649	0.0783
TNCF_88M	YES	0.0177	0.0188
TNCF_101	NO	0.1535	0.0999
TNCF_105	NO	2.3635	2.2542
TNCF_106	NO	0.7177	0.5608
TNCF_109	NO	1.262	1.63
TNCF_130	NO	1.4477	1.366
TNCF_133	NO	0.0336	0.0598
TNCF_133_1	NO	0.0086	0.0119
TNCF_151	NO	4.4742	2.2776
TNCF_151M	YES	0.0176	0.0087
TNCF_154	NO	0.1406	0.1426
TNCF_155	NO	0.0046	0.0124
TNCF_155_1	NO	0.9051	1.1274
TNCF_165	NO	0.0113	0.0257
TNCF_167	NO	0.1702	0.2495
TNCF_167_1	NO	19.7247	19.1355
TNCF_174	NO	0.0892	0.1629
TNCF_175	NO	0.2394	0.3112
TNCF_176	NO	0	0
