# Supplementary material for: Persistence and Microevolution of Pseudomonas aeruginosa in the Cystic Fibrosis Lung: A Single-Patient Longitudinal Genomic Study
Source: Front Microbiol. 2019 Jan 11;9:3242. doi: 10.3389/fmicb.2018.03242 (PMC6340092; doi:10.3389/fmicb.2018.03242)
Supplement: Supplementary file 14 [file Table_5.pdf]

Additional file 13: Table S4. Correlation analysis between phenotypes. The upper diagonal of the table shows the rho values (grey cells), while the lower diagonal shows the p-values (green cells).

	MDR	Mucoidy	Biofilm
MDR	-	-0.77	0.65
Mucoidy	0	-	-0.47
Biofilm	0	0.002	-
