# Supplementary material for: Persistence and Microevolution of Pseudomonas aeruginosa in the Cystic Fibrosis Lung: A Single-Patient Longitudinal Genomic Study
Source: Front Microbiol. 2019 Jan 11;9:3242. doi: 10.3389/fmicb.2018.03242 (PMC6340092; doi:10.3389/fmicb.2018.03242)
Supplement: Supplementary file 15 [file Table_6.pdf]

Additional file 15: Table S6. Variants present in the population in genes related to antibiotic-resistant itolates.

Gene	3	4M	6	7M	10M	12	13	14	16	23	23M	32	32M	42	42M	49M	68	69	76	85	88M	101	105	106	109	130	133	133-1	151	151M	154	155	155-1	165	167	167-1	174	175	176	Variation
	C	C	C	C	C	C	C	C	C	C	C	C	C	C	C	C	C	C	C	C	C	C	C	C	C	C	C	C	T	C	C	C	C	C	C	C	C	C	C	P_{276}-S
	T	G	T	G	G	T	T	T	G	G	G	G	G	G	G	G	T	T	T	T	T	T	T	T	T	T	T	T	T	G	T	T	T	T	T	T	T	T	T	E_{468}-D
gyrB	A	A	A	A	A	A	A	A	A	A	A	A	A	A	A	A	A	A	A	A	A	A	A	A	A	A	A	A	A	A	A	A	A	A	G	A	G	G	A	B_{620}-G
	G	G	G	G	G	G	G	G	G	G	G	G	G	G	G	G	G	G	G	G	G	G	G	G	G	G	G	G	G	G	G	G	G	G	G	G	A	A	G	G_{630}-D
	C	C	C	C	T	C	C	C	C	T	T	T	T	T	T	T	C	C	C	C	C	C	C	C	C	C	C	C	C	C	C	C	C	C	C	C	C	C	C	S_{749}-P
parC	A	A	A	-	A	A	A	A	A	A	A	A	A	A	A	A	A	A	A	A	A	A	A	A	A	A	A	A	A	A	A	A	A	A	A	A	A	A	A	N_{170}-S
	C	T	C	T	T	C	T	C	T	T	T	T	T	T	T	T	C	C	C	T	T	T	C	C	C	C	C	T	C	T	C	C	C	C	C	C	C	C	C	P_{360}-L
	G	G	G	G	G	G	G	G	G	G	G	G	G	G	G	G	G	G	G	G	G	G	G	G	G	G	A	G	G	G	G	G	G	G	G	G	G	G	G	L_{455}-F
parE	T	T	T	T	T	T	T	T	T	T	T	T	T	T	T	T	T	C	T	T	T	T	C	C	T	T	C	T	T	T	T	C	T	T	C	C	C	C	C	H_{59}-R
	G	G	G	G	G	G	G	G	G	G	G	G	G	G	G	G	G	A	G	G	G	G	A	A	G	G	A	G	G	G	G	A	G	G	A	A	A	A	A	R_{30}-C
oprD	-	C	-	C	C	-	C	-	C	C	C	C	C	C	C	C	-	C	-	C	C	-	-	-	C	C	-	C	C	C	C	-	C	-	-	-	-	-	-	frameshift
	G	G	G	G	G	G	G	G	G	G	G	G	G	G	G	G	G	G	G	G	G	G	G	C	G	G	G	G	G	G	G	G	G	G	G	G	G	G	G	R_{86}-P
mexG	T	C	C	C	C	T	C	C	C	C	C	C	C	C	C	C	T	C	T	T	T	C	T	T	T	T	T	C	T	C	C	T	C	T	T	T	T	T	T	P_{51}-S
