# Supplementary material for: Persistence and Microevolution of Pseudomonas aeruginosa in the Cystic Fibrosis Lung: A Single-Patient Longitudinal Genomic Study
Source: Front Microbiol. 2019 Jan 11;9:3242. doi: 10.3389/fmicb.2018.03242 (PMC6340092; doi:10.3389/fmicb.2018.03242)

Additional file 7: Figure S5: Heatmap of TA genes.
Abundance of TA genes in the population, both isolates genomes and genes were clustered using the complete-linkage method.

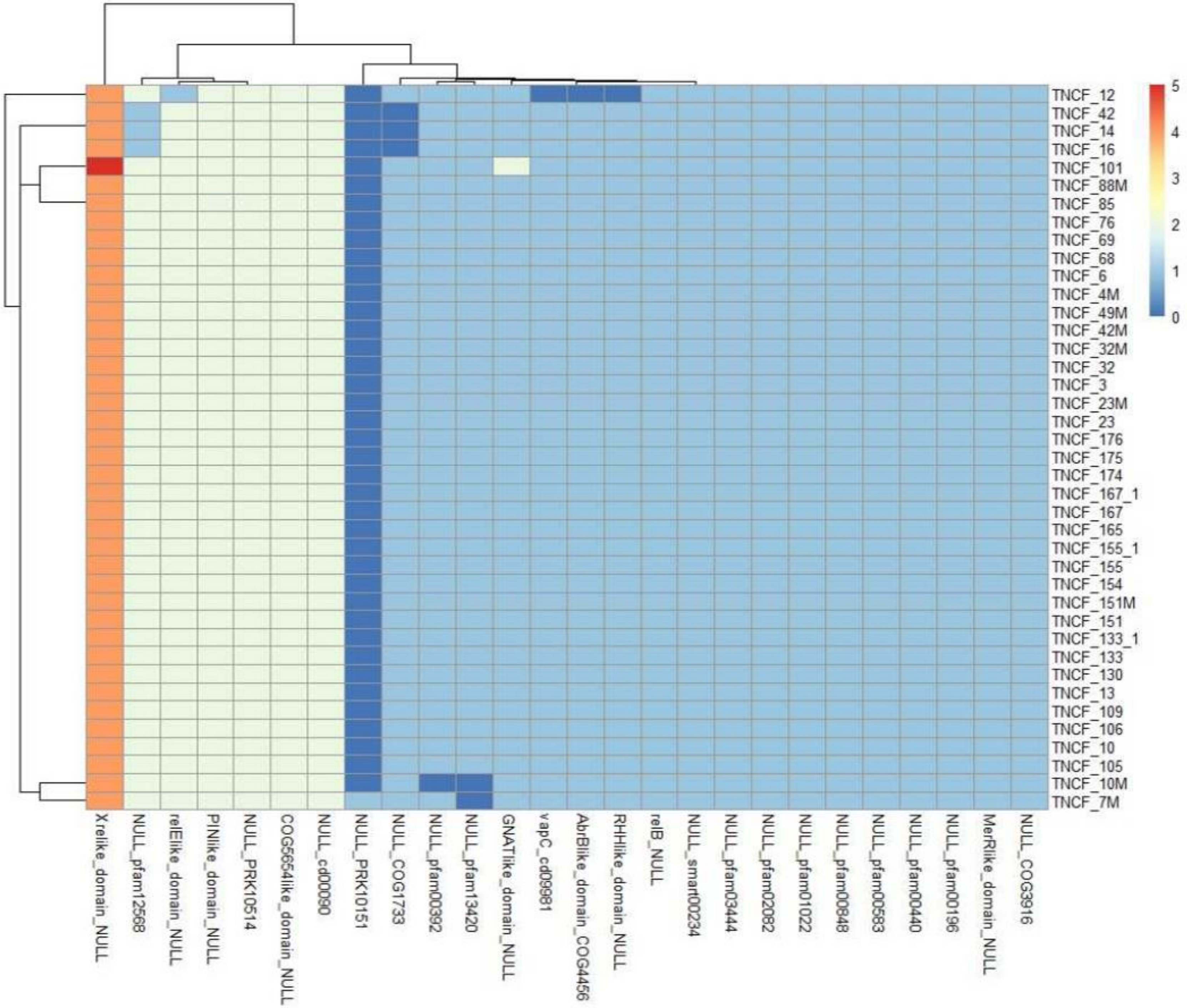

Supplement: Supplementary file 5 [file Image_5.pdf]
